# Performance Evaluation of Bayesian Network Learning Algorithms in Structural Equation Modeling: A Simulation Study

**DOI:** 10.3390/e28090955

**Published:** 2026-08-25

**Authors:** Tugay Karadag

**Affiliations:** Department of Statistics, Yildiz Technical University, Istanbul 32220, Türkiye; karadagt@yildiz.edu.tr

**Keywords:** Bayesian network learning, causal discovery, hybrid algorithms, simulation study, structural equation modeling

## Abstract

Bayesian Network (BN) learning algorithms may exhibit substantially different performance across graph structures, sample sizes, and evaluation criteria. Comparative evidence on BN learning under structurally validated conditions remains limited. This study evaluates BN learning algorithms for causal discovery through a simulation-based framework that integrates multiple graph structures, sample sizes, and structural equation modeling (SEM)-based validation within a common design. Fourteen constraint-based, score-based, and hybrid algorithms were examined across five randomly generated directed acyclic graphs (DAGs) containing latent constructs and four sample sizes (*n* = 200, 500, 1000, and 2500). For each DAG–sample size combination, 1000 datasets were generated and validated using SEM, yielding 20,000 accepted datasets. Performance was assessed primarily by Matthews correlation coefficient (MCC), supported by directed structural Hamming distance (SHD) and F1. The results reveal substantial variation across DAG structures, sample sizes, and evaluation metrics, with mean MCC ranging from −0.43 to 0.65. Peter–Clark Stable achieved the strongest performance in several conditions, whereas hybrid algorithms were frequently among the weaker performers. Increasing sample size did not produce uniform performance gains, and no algorithm or algorithm class consistently dominated across all settings. These findings show that BN structure learning performance is strongly structure- and condition-dependent and support evaluation across multiple controlled DAG configurations.

## 1. Introduction

Understanding causal relationships from data has traditionally relied on trial-and-error approaches or prior knowledge supported by existing literature. However, in cases where such prior information or references are insufficient, approaches that extract causal relationships directly from raw data have gained significant attention in the literature [1]. Learning causal relationships from empirical data offers substantial advantages to researchers. Nevertheless, while constraint-based algorithms are generally faster than score-based algorithms, their restrictive assumptions remain a key limitation [2]. The inference of causality through empirical data has been well-documented in various domains, including statistics [3,4], econometrics [5], computer science [6], and information systems [7].

Despite the increasing number of Bayesian Network (BN) learning approaches and comparative studies, evidence regarding their relative performance remains fragmented across different algorithms, structural configurations, sample sizes, and evaluation criteria. Existing comparisons often focus on specific algorithm sets, limited structural settings, or particular performance measures, making it difficult to determine whether reported performance advantages generalize across different underlying DAG structures. This creates a need for systematic comparisons in which multiple algorithms are evaluated under varying structural and sample size conditions using complementary performance criteria.

This study distinguishes itself from previous research by using 14 different BN learning algorithms and employing a systematic simulation-based approach. Specifically, it utilizes multiple simulated datasets across four different sample sizes and five randomly generated DAGs with latent constructs. Each dataset was generated based on predefined structural configurations and analyzed using structural equation modeling (SEM). For each analysis, the significance of coefficients and the validity of goodness-of-fit indices were ensured, leading to a large collection of validated SEM analyses under controlled conditions. Algorithm performance was evaluated using multiple performance metrics, with MCC used as the primary metric and SHD and F1 as supporting measures. The contributions of the study are as follows.

(1) It makes a comprehensive algorithm comparison by evaluating the performance of 14 different BN learning algorithms, offering a thorough comparison that extends beyond existing literature.
(2) It employs a simulation-based approach across varying sample sizes and multiple DAGs with latent constructs, allowing algorithm performance to be examined under diverse yet controlled conditions.
(3) It utilizes datasets generated from predefined and structurally valid latent DAGs that are subsequently validated through SEM, ensuring both the significance of coefficients and the appropriateness of goodness-of-fit indices. This approach provides a controlled and trustworthy framework for evaluating algorithm performance.
(4) It addresses a gap in the literature by offering novel insights into how BN learning algorithms perform across different underlying structural DAGs, thereby informing both methodological research and practical applications.

## 2. Related Work

A wide range of methodological and computational approaches has been developed for causal discovery, including conditional-probability-based methods [8,9,10], TETRAD [11], and widely used implementations such as pcalg, bnlearn, causal-learn, and benchpress [1,12,13,14]. Zheng and Pavlou [8] also proposed the Bayesian Networks for Latent Variables (BN-LV) approach, which integrates measurement and structural model discovery.

Although previous studies have compared causal discovery algorithms using both simulation and empirical datasets, comprehensive evidence on the conditions under which different algorithms perform well remains limited. Earlier comparative studies have reported different leading algorithms depending on the methods, datasets, and structural conditions considered [15,16,17,18]. In particular, Singh, Gupta, Tewari and Shroff [18] showed that algorithm performance may deteriorate as the number of latent variables increases. More recently, Farnia et al. [19] demonstrated that causal structure learning performance is strongly affected by latent confounding and structural characteristics, further illustrating that algorithm effectiveness is context-dependent.

The application of Bayesian Networks to causal inference is well-established in the literature [20,21,22]. Constantinou et al. [23] evaluated 15 Bayesian Network algorithms on real datasets, while Kitson et al. [24] emphasized the enduring competitiveness of Hill-Climbing, Peter–Clark, and Greedy Equivalence Search algorithms. Kitson and Constantinou [25] further showed that variable ordering can substantially affect structure learning performance and that the magnitude of this sensitivity varies across underlying networks. Beyond individual algorithm comparisons, evaluation outcomes may also be sensitive to the choice of performance metrics. Velev and Lessmann [26] used a multidimensional evaluation framework and showed substantial performance variation across experimental conditions, supporting the use of complementary criteria in comparative evaluations.

More recent studies have also focused on systematic and large-scale evaluations of structure learning methodologies. A comprehensive review by He et al. [27] categorized BN learning algorithms into constraint-based, score-based, and hybrid approaches, and highlighted the lack of standardized evaluation protocols and large-scale comparative benchmarks in the literature. Comparative evidence also indicates that methodological choices such as scoring criteria can substantially influence the learned structure [28]. Hyperparameter selection represents another source of variability. Chobtham and Constantinou [29] found that tuning can improve F1 and SHD under some conditions, but does not consistently improve graphical accuracy, while default settings can provide a strong baseline.

Several studies have explored algorithm comparisons within specific methodological paradigms. Yu et al. [30] investigated local-to-global BN structure learning approaches and showed that feature selection-based methods can achieve competitive structural accuracy while significantly improving computational efficiency when compared with existing algorithms. Similarly, Wang et al. [31] evaluated multiple local BN structure learning algorithms in high-dimensional settings, reporting comparative performance primarily in terms of F1 score and highlighting the challenges of reliable structure recovery as dimensionality increases. Recent methodological studies have continued to propose hybrid and alternative structure learning strategies aimed at improving accuracy and computational efficiency [32]. However, limitations related to local optima and computational complexity remain relevant. The continuing need for controlled benchmarking has also been emphasized by Göbler et al. [33], who noted the difficulty of validating causal discovery methods when the true causal structure of real-world data is unknown.

As summarized in Table 1, previous studies have examined BN and causal structure learning under varying structural, sample size, methodological, and evaluation conditions. However, these comparisons generally differ in their algorithm sets, experimental designs, and evaluation frameworks. The present study addresses this fragmented evidence by evaluating 14 BN learning algorithms across five randomly generated DAGs with latent constructs and four sample sizes within a common simulation framework, with the generated datasets additionally validated through SEM. This unified design enables structure- and sample size-dependent algorithm performance to be evaluated under consistent conditions.

## 3. Materials and Methods

### 3.1. Simulation Data

**Figure 1:**
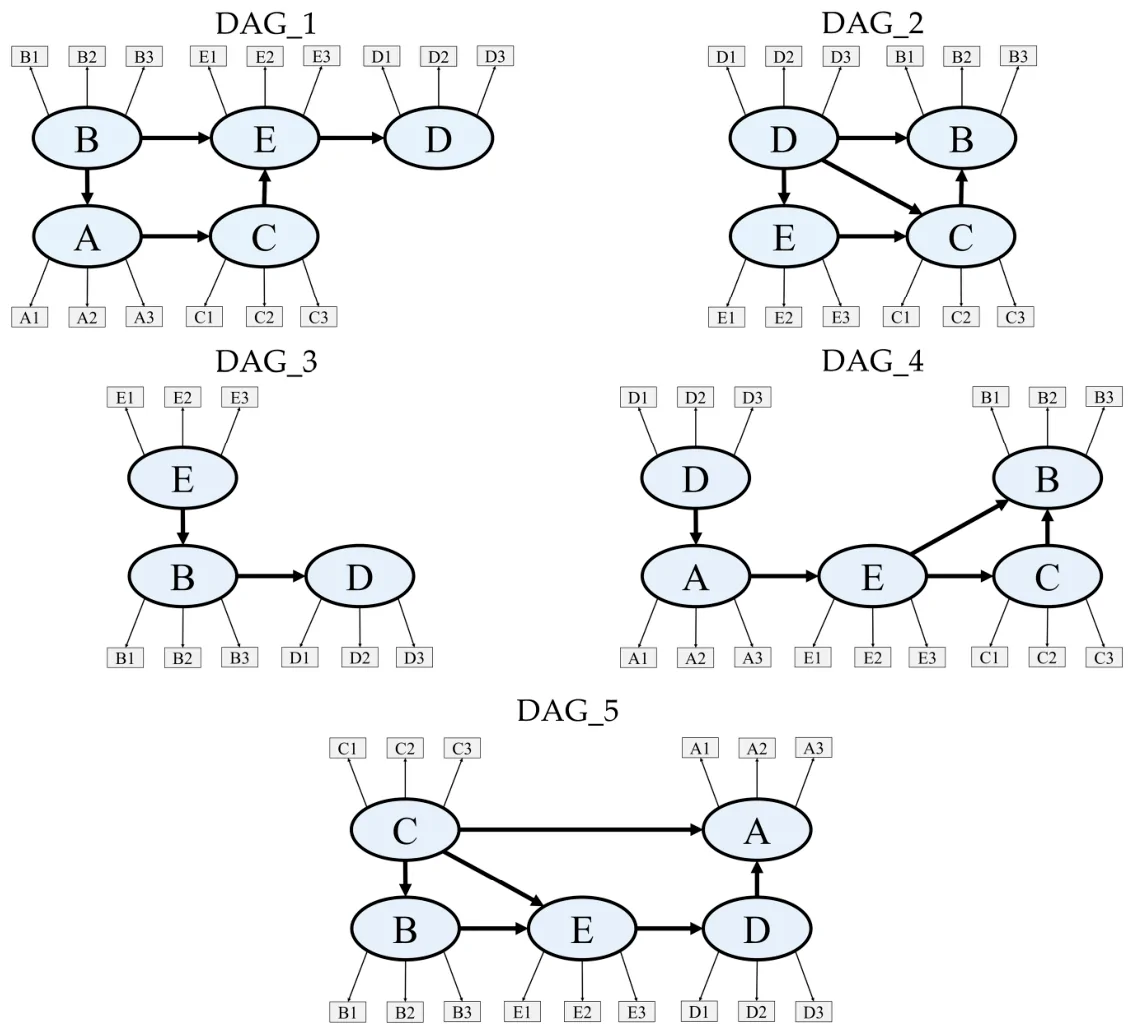
Randomly generated DAGs with latent constructs (three indicators per latent variable). A–E denote latent constructs, while A1–A3, B1–B3, C1–C3, D1–D3, and E1–E3 denote their corresponding observed indicators.

For each sample size (*n* = 200, *n* = 500, *n* = 1000, and *n* = 2500), 1000 different datasets were generated for each randomly generated DAGs, resulting in a large collection of simulated datasets across multiple structural configurations. These datasets were created based on randomly generated but fixed DAGs with latent constructs, as given in Figure 1. Each dataset was analyzed using SEM to ensure the significance of the coefficients and the validity of the structural model.

Descriptive statistics and goodness-of-fit results for all simulated datasets are reported in the Supplementary Materials. The analyses showed that all coefficients were statistically significant; therefore, their statistical results are not included in the table.

When examining the goodness-of-fit indices, it was observed that across all DAG structures and sample sizes, the fit indices met or exceeded the recommended threshold values. For instance, the Standardized Root Mean Squared Residual (SRMR) values were below the recommended threshold of 0.05 (values below 0.10 are also considered acceptable) [34]. The Goodness-of-Fit Index (GFI) values exceeded the acceptable threshold of 0.90 [34]. Similarly, the Adjusted GFI (AGFI) values were above 0.90. The Comparative Fit Index (CFI) values were well above the recommended threshold of 0.95 [35]. Other indices, such as Normed Fit Index (NFI), Non-Normed Fit Index (NNFI), and Incremental Fit Index (IFI), also exceeded the recommended threshold values [34]. Finally, the Root Mean Square Error of Approximation (RMSEA) values were well below the recommended threshold of 0.06 [35,36], and the Relative Chi-Squared values were far below the recommended threshold of 3 [34]. These findings indicate that all datasets are valid and suitable for further analyses.

### 3.2. Simulation Setting and Data-Generating Process

The simulation design is based on randomly generated DAGs with latent constructs, with a maximum of five latent variables (A, B, C, D, and E). The DAGs were randomly generated under connectivity and acyclicity constraints, with the number of latent variables varying between three and five. This upper bound was deliberately selected to maintain interpretability of the structural models and to avoid excessive complexity in the combined SEM and BN learning framework. Accordingly, the study focuses on relatively small causal structures and does not aim to evaluate scalability in high-dimensional networks. In addition, to avoid complexity, the number of indicator variables for each latent variable were chosen as three. Within each DAG structure, the underlying relations among latent variables were fixed across all replications and sample sizes.

The five randomly generated DAGs differ in their connectivity patterns and in the number and arrangement of directed relationships, thereby providing structurally distinct scenarios for evaluating algorithm performance. These differences allow the algorithms to be evaluated across multiple structural configurations rather than under a single fixed topology.

Structural equations followed a linear additive form, where each endogenous latent variable was generated as a weighted sum of its parent latent variables plus an error term. Structural path coefficients were fixed at 0.6 and 0.4, depending on the corresponding relationship, and remained unchanged across replications and sample sizes to maintain consistent signal strength across simulation conditions. Indicator loadings were fixed at 0.8, with corresponding residual variances set to 0.36. Errors were generated independently, and (unless otherwise stated) a homoskedastic Gaussian noise structure was assumed. Latent-variable variances were fixed at 1 across the simulated models.

To provide a single manifest variable per latent construct for the Bayesian network learning stage, the three indicators of each latent variable were aggregated by their mean and the algorithms were applied to these aggregated variables. This averaging reduces the number of variables entered into the BN algorithms and provides a single score for each latent construct. However, averaging may also hide some differences among the individual indicators. The simulation assumed causal sufficiency (i.e., no unmeasured common causes beyond the variables included in the model) and faithfulness between the data-generating distribution and the underlying DAG.

Although the underlying causal structure among the latent variables is fully specified in the simulation design, the study intentionally includes a measurement stage with multiple observed indicators per latent construct. This choice reflects common empirical practice in SEM, where latent constructs are not directly observed and are often represented by composite scores derived from multiple indicator variables. By aggregating these indicator variables prior to BN learning, the study evaluates algorithmic performance under conditions that more closely resemble applied research settings, rather than idealized fully observed latent structures. The additional SEM validation step serves to confirm that the measurement and aggregation process preserves the intended structural relationships before causal discovery is performed.

The simulation followed a Monte Carlo design with 1000 accepted datasets for each DAG–sample size combination. The Monte Carlo approach was selected because it enables repeated evaluation of BN learning algorithms under controlled data-generating conditions in which the true underlying DAG is known, allowing both average performance and variability across replications to be assessed. Candidate datasets were generated iteratively and retained only when the fitted SEM satisfied the predefined model-fit and structural-path significance criteria. Fixed random seeds were used for DAG generation, whereas replication-level seeds for the simulated datasets were not stored.

All simulations and analyses were conducted on a computer equipped with an 11th Gen Intel Core i7-1165G7 processor at 2.80 GHz and 32 GB RAM, running 64-bit Windows 11 and R version 4.2.2.

### 3.3. Algorithms

The algorithms in the study are categorized into three distinct classes: constraint-based, score-based, and hybrid. Constraint-based algorithms use conditional independence tests to extract conditional independence constraints from the data while score-based algorithms rank the network structures based on a goodness-of-fit score and produce the optimal result. Hybrid algorithms combine the features of both approaches, offering a hybrid methodology. All algorithms were implemented using the bnlearn package and were performed with their default hyperparameter settings in the primary analysis. This setup was made to ensure comparability across methods and to reflect typical out-of-the-box usage in applied settings. An additional algorithm-specific hyperparameter tuning analysis was conducted as a sensitivity analysis, as described in Section 3.4.

#### 3.3.1. Constraint-Based Algorithms

- Peter–Clark Stable (PCS): The PCS algorithm, derived from the names of its developers Peter Spirtes and Clark Glymour, is among the most commonly applied constraint-based algorithms in causal discovery [37]. The PCS algorithm is an enhanced version of the original PC algorithm, designed for causal discovery. It begins by identifying pairs of variables connected by an undirected arc, determined by conditional independence tests across increasing subsets of variables. Next, it detects v-structures (specific directional relationships) among non-adjacent nodes with a shared neighbor, using separating sets identified earlier. Finally, the remaining arc directions are determined with logical rules to construct the completed partially directed acyclic graph (CPDAG), that represents the equivalence class of possible causal graphs [24].
- Grow-Shrink (GS): It is a method for learning the Markov Blanket of a target variable. It operates in two stages: growing the blanket by adding relevant variables and shrinking it by removing false positives [38].
- Incremental association Markov Blanket (IAMB): It identifies the Markov Blanket of a target variable using a two-phase process. First, it applies a forward selection step to include relevant variables. Then, it removes false positives in a pruning phase, ensuring a more accurate result.
- Fast-IAMB: It improves on IAMB by reducing the number of conditional independence tests through speculative forward selection. It is particularly suited for large datasets where computational cost is a concern [39].
- Interleaved IAMB (Inter-IAMB): This algorithm is a version of IAMB that gradually interleaves the forward selection. This approach reduces the likelihood of including false positives during the Markov Blanket identification process [39].
- IAMB with False Discovery Rate Correction (IAMB-F): It is also a different version of IAMB and adjusts the threshold of significance of tests by false discovery rate.
- Max–Min Parents and Children (MMPC): This algorithm determines neighbors through a forward selection approach, optimizing the minimum association measure observed within any subset of nodes chosen during earlier steps [40].
- Semi-Interleaved HITON-PC (SI-HITON-PC or SPC): This algorithm is developed by combining the HITON approach [41] with the features of the PC algorithm. This integration enhances the algorithm’s ability to effectively identify causal relationships and dependencies within the data. For further details, see Aliferis et al. [42].
- Hybrid Parents and Children (HPC): Building on the IAMB-F framework, it identifies the parents and children of each variable in a network, similar to MMPC and SI-HITON-PC [19].

#### 3.3.2. Score-Based Algorithms

- Hill-Climbing (HC): It explores the search space of DAGs by iteratively adding, removing, or reversing edges to maximize a scoring function. It is prone to local optima, but random restarts can mitigate this limitation.
- Tabu search (Tabu): It improves upon hill-climbing by keeping a “tabu list” of previously visited states to avoid cycling. This allows it to escape local optima, making it effective for large-scale network structure learning tasks.

#### 3.3.3. Hybrid Algorithms

- Max–Min Hill-Climbing (MMHC): It combines two algorithms: (1) MMPC, which narrows the search space by identifying relevant variables; (2) HC, which optimizes the network structure within the defined space.
- Hybrid HPC (H2PC): It combines two algorithms: (1) HPC; (2) HC.
- Restricted Maximization (RSMAX2): It is a versatile extension of the Sparse Candidate method. It allows for combining any combination of constraint and score-based techniques.

### 3.4. Performance Metrics

The evaluation of the algorithms is based on metrics derived from the confusion matrix given in Table 2, which summarizes outcomes as True Positive (TP), True Negative (TN), False Positive (FP), and False Negative (FN). We can define TP, TN, FP, and FN in this study as follows: When the simulated data indicates a direct relationship from X to Y (actual value is +) and the algorithm correctly predicts the same (predicted value is +), this is counted as a TP, whereas if the simulated data indicates there is no direct relationship from X to Y (actual value is −) and the algorithm correctly predicts no relationship (predicted value is −), this is counted as a TN. Conversely, when the simulated data indicates there is no direct relationship from X to Y (actual value is −) but the algorithm incorrectly predicts the presence of a relationship (predicted value is +), this is counted as a FP. Finally, if the simulated data indicates a direct relationship from X to Y (actual value is +) but the algorithm fails to predict it (predicted value is −), this is counted as a FN.

Because several BN learning algorithms return undirected or partially directed edges, such edges were handled explicitly in the evaluation process. An undirected edge between two variables (X–Y) was treated as representing two possible directed relationships, X → Y and Y → X. If the true underlying relationship was X → Y and the learned graph contained an undirected edge X–Y, the X → Y direction was counted as a true positive, while the reverse direction Y → X was counted as a false positive. This rule was consistently applied when computing TP, FP, FN, and TN based on directed edge comparisons. From these outcomes, various performance metrics can be calculated to assess the success levels of algorithms. Accuracy, sensitivity or recall, precision, specificity, F1 score, Cohen’s kappa, and Matthews correlation coefficient metrics are used in this study to compare the performances of the algorithms. In addition to these classification-based metrics, the directed Structural Hamming Distance (SHD) is also calculated. For the main comparative assessment, MCC was designated as the primary metric, while SHD and F1 were used as supporting measures; the remaining metrics were retained to provide additional metric-specific information.

An algorithm that performed best in one metric might not be the best in another. To provide a complementary summary of overall performance, an overall rank was calculated. Each algorithm was ranked for every performance metric, where the best algorithm received a rank of 1, and the worst received a rank of 14. After ranking, the average rank across all metrics was calculated for each algorithm, and this average was reported as the overall rank. Overall rank was therefore treated as a complementary summary rather than as an additional performance metric. However, all performance metrics were also analyzed and interpreted individually to ensure a detailed and metric-specific evaluation.

Accuracy indicates the ratio of correctly classified instances among all cases. Sensitivity (or Recall) evaluates the ability to correctly identify positive cases, while precision calculates the proportion of TP predictions out of all predicted positive instances. Specificity assesses the ability to correctly classify negative cases, highlighting the model’s performance in avoiding FPs. The F1 score is the harmonic mean of precision and sensitivity, offering a balanced measure that considers both FP and FN. Cohen’s kappa measures the level of agreement between predicted and actual classifications, adjusting for chance-based agreement. Matthews correlation coefficient (MCC) provides a balanced measure of classification quality, considering all elements of the confusion matrix. Finally, SHD measures the accuracy of the learned structure by quantifying the differences between the predicted edges and the true relationships in the original model [16]. In this study, directed SHD was calculated as the sum of extra edges (EE) and missing edges (ME). A reversed relationship therefore contributes one EE and one ME. EE can also be referred to as false positive edges. These are edges that are inferred by the algorithm but actually do not exist in the original structure. ME correspond to true edges in the original structure that are not obtained by the algorithm, meaning that an existing relationship is incorrectly omitted in the learned graph [43].

For these metrics, the higher value means the better performance, whereas for SHD the situation is the opposite. The equations regarding these metrics with their abbreviations are given in Table 3.

As a sensitivity analysis, algorithm-specific hyperparameter tuning was additionally performed, with 300 datasets from each DAG–sample size condition used for parameter selection and the remaining 700 reserved for independent evaluation, to assess whether the comparative findings depended on the default settings. Computational efficiency was additionally assessed using elapsed solution time under the same hardware and software environment. For each algorithm and DAG–sample size condition, the default configuration was applied and the analysis was repeated three times; the median elapsed time was used to calculate mean solution time per dataset. As a cross-software sensitivity analysis, the PC algorithm was additionally applied to the same simulated datasets using TETRAD and compared with the corresponding bnlearn implementation, with Fisher’s Z test (α = 0.05), unlimited conditioning depth, stable FAS, and Sepsets-based collider orientation used to provide a comparable configuration.

## 4. Results

This section presents the performance of the BN learning algorithms across five randomly generated DAGs with latent constructs given in Figure 1. To maintain readability, the main-text tables present the *n* = 200 results as a common baseline for algorithm comparison, while results for the other sample sizes are discussed in the manuscript and reported in detail in the Supplementary Materials.

Table 4, Table 5, Table 6, Table 7 and Table 8 report the performance of the algorithms for DAG_1 through DAG_5, respectively. For clarity, the main-text tables report MCC as the primary performance metric, together with SHD and F1 as supporting measures, while the overall rank is retained as a complementary summary of performance across all evaluated metrics. The complete metric-specific results for *n* = 200 are provided in Appendix A, while the corresponding detailed results for the remaining sample sizes are available in the Supplementary Materials. Mean metric values are given along with the standard deviations in parentheses. The “R” values displayed next to each metric represent the ranking of the algorithms based on that metric, while the last column shows the average rank across all metrics.

The results for DAG_1 with *n* = 200 are given in Table 4. According to the primary metric, MCC, HPC achieved the best performance (0.55), followed by MMPC and SPC (0.54), while PCS obtained an MCC of 0.51. Regarding the supporting measures, PCS achieved the lowest SHD (4.4), while HPC and PCS obtained the highest F1 value (0.65). HC showed the weakest performance according to MCC (0.23), SHD (5.9), and F1 (0.48). When overall ranking is considered, PCS and HPC emerge as the two best-performing algorithms for DAG_1, while Tabu and HC rank as the weakest methods.

When the outputs for other sample sizes are examined in the Supplementary Document, PCS generally shows stronger performance at the larger sample sizes in terms of the primary metric, MCC. Its MCC increases from 0.51 at *n* = 200 to 0.65 at *n* = 500, 0.64 at *n* = 1000, and 0.57 at *n* = 2500. In contrast, HPC—one of the strongest algorithms at *n* = 200—exhibits a substantial performance decline as *n* increases. Specifically, its MCC decreases from 0.55 at *n* = 200 to 0.39 at *n* = 2500. This reversal suggests that HPC is sensitive to sample size growth within DAG_1, particularly in terms of overall structural recovery. The supporting F1 and SHD results reported in the Supplementary Document generally support this pattern. PCS shows improved F1 performance as *n* increases, whereas the SHD performance of HPC deteriorates at larger sample sizes. Considering overall rankings across all sample sizes, PCS consistently ranks first, while HC remains the weakest algorithm.

**Table 4:** Selected performance metrics and overall ranking of BN learning algorithms for DAG_1 with sample size *n* = 200.

	MCC	R_MCC_	SHD	R_SHD_	F1	R_F1_	Overall Rank
**HPC**	0.55 (0.06)	1	5.4 (0.82)	3	0.65 (0.04)	1	3.1
**MMPC**	0.54 (0.07)	2	5.5 (0.91)	5	0.64 (0.04)	3	4.7
**SPC**	0.54 (0.07)	3	5.5 (0.91)	4	0.64 (0.04)	4	4.8
**PCS**	0.51 (0.21)	4	4.4 (1.9)	1	0.65 (0.15)	2	3.1
**F-IAMB**	0.51 (0.12)	5	5.6 (0.99)	12	0.62 (0.08)	5	7.9
**IAMB**	0.51 (0.13)	6.5	5.6 (0.99)	10.5	0.62 (0.08)	6.5	7.8
**I-IAMB**	0.51 (0.13)	6.5	5.6 (0.99)	10.5	0.62 (0.08)	6.5	7.8
**GS**	0.50 (0.13)	8	5.5 (0.98)	8	0.62 (0.09)	8	7.9
**IAMB-F**	0.48 (0.14)	9	5.3 (0.80)	2	0.61 (0.10)	9	6.4
**H2PC**	0.27 (0.41)	10	5.5 (3.1)	6	0.50 (0.28)	11	8
**MMHC**	0.27 (0.41)	11	5.5 (3.1)	7	0.50 (0.28)	12	9.1
**RSMAX2**	0.27 (0.41)	12	5.5 (3.1)	9	0.50 (0.28)	10	9.9
**Tabu**	0.26 (0.38)	13	5.7 (2.9)	13	0.49 (0.25)	13	11.8
**HC**	0.23 (0.41)	14	5.9 (3.2)	14	0.48 (0.28)	14	12.9

Values in parentheses indicate standard deviations. F-IAMB: Fast-IAMB; GS: Grow-Shrink; H2PC: Hybrid HPC; HC: Hill-Climbing; HPC: Hybrid Parents and Children; IAMB: Incremental association Markov Blanket; IAMB-F: IAMB with False Discovery Rate Correction; I-IAMB: Interleaved IAMB; MMHC: Max–Min Hill-Climbing; MMPC: Max–Min Parents and Children; PCS: Peter–Clark Stable; RSMAX2: Restricted Maximization; SPC: Semi-Interleaved HITON-PC; Tabu: Tabu search. MCC: Matthews Correlation Coefficient; SHD: Directed Structural Hamming Distance; F1: F1 Score; R: Rank; Overall Rank is based on the rankings across all evaluated performance metrics; the complete metric-specific results are provided in Appendix A.

The results for DAG_2 with *n* = 200 observations are presented in Table 5. For DAG_2, IAMB-F has the best performance according to the primary metric, MCC (0.36). IAMB-F also ranks first according to both supporting measures, with an SHD of 4.6 and an F1 score of 0.65. Based on overall rankings, IAMB-F clearly outperforms all other algorithms for DAG_2, whereas HC and the hybrid algorithms rank among the weakest.

When results for different sample sizes are examined in the Supplementary Document, the MCC values of several algorithms converge as *n* increases. HPC increases from 0.30 at *n* = 200 to 0.38 at *n* = 500 and remains at 0.38 for *n* = 1000 and *n* = 2500. In contrast, HC consistently produces the weakest performance across all sample sizes. In addition, the performance of IAMB-F decreases as *n* grows, with its MCC declining from 0.36 at *n* = 200 to 0.28 at *n* = 2500.

For the supporting F1 and SHD measures reported in the Supplementary Document, HPC produces particularly strong results from *n* = 1000 onward. Conversely, Tabu, HC, and the hybrid algorithms consistently generate relatively weak results for these supporting measures. A similar pattern is observed for the overall ranking, which is retained as a complementary summary measure. These findings indicate that HPC exhibits strong robustness in the supporting measures and overall ranking for DAG_2, particularly at larger sample sizes.

A remarkable finding for DAG_2 is the substantial improvement of PCS as the sample size increases. This pattern is particularly clear for MCC. For example, for DAG_2, the MCC value of PCS increases from 0.01 at *n* = 200 to 0.38 at *n* = 2500. In contrast, several other algorithms do not show a comparable improvement with increasing sample size.

**Table 5:** Selected performance metrics and overall ranking of BN learning algorithms for DAG_2 with sample size *n* = 200.

	MCC	R_MCC_	SHD	R_SHD_	F1	R_F1_	Overall Rank
**IAMB-F**	0.36 (0.21)	1	4.6 (1.4)	1	0.65 (0.11)	1	2.4
**IAMB**	0.34 (0.17)	2.5	5.0 (1.1)	3	0.65 (0.08)	2.5	3.7
**I-IAMB**	0.34 (0.17)	2.5	5.0 (1.1)	3	0.65 (0.08)	2.5	3.7
**GS**	0.34 (0.17)	4	5.0 (1.1)	3	0.65 (0.08)	5	4.4
**F-IAMB**	0.34 (0.17)	5	5.0 (1.1)	5	0.65 (0.08)	4	5.3
**MMPC**	0.31 (0.12)	6	5.2 (0.64)	7.5	0.64 (0.04)	6	7.1
**SPC**	0.31 (0.12)	7	5.2 (0.64)	7.5	0.64 (0.04)	7	7.4
**HPC**	0.30 (0.12)	8	5.2 (0.58)	6	0.64 (0.04)	8	7.3
**PCS**	0.01 (0.31)	9	6.4 (1.5)	9	0.50 (0.15)	9	9.4
**Tabu**	−0.34 (0.31)	10	7.8 (1.8)	10	0.30 (0.16)	10	8.9
**MMHC**	−0.40 (0.25)	11	8.1 (1.4)	12	0.27 (0.12)	13	10.7
**RSMAX2**	−0.40 (0.25)	12	8.1 (1.4)	13	0.27 (0.12)	12	11.4
**H2PC**	−0.40 (0.25)	13	8.1 (1.4)	11	0.27 (0.12)	11	11
**HC**	−0.41 (0.26)	14	8.2 (1.5)	14	0.27 (0.12)	14	12.5

Values in parentheses indicate standard deviations. F-IAMB: Fast-IAMB; GS: Grow-Shrink; H2PC: Hybrid HPC; HC: Hill-Climbing; HPC: Hybrid Parents and Children; IAMB: Incremental association Markov Blanket; IAMB-F: IAMB with False Discovery Rate Correction; I-IAMB: Interleaved IAMB; MMHC: Max–Min Hill-Climbing; MMPC: Max–Min Parents and Children; PCS: Peter–Clark Stable; RSMAX2: Restricted Maximization; SPC: Semi-Interleaved HITON-PC; Tabu: Tabu search. MCC: Matthews Correlation Coefficient; SHD: Directed Structural Hamming Distance; F1: F1 Score; R: Rank; Overall Rank is based on the rankings across all evaluated performance metrics; the complete metric-specific results are provided in Appendix A.

The results for DAG_3 with *n* = 200 observations are given in Table 6. Due to the relatively small number of latent variables and structural relations in DAG_3, many algorithms yield highly similar performance values. For *n* = 200, according to MCC, F-IAMB, GS, HPC, IAMB, I-IAMB, MMPC, and SPC produce the highest and identical value (0.50), while H2PC, HC, MMHC, RSMAX2, and Tabu yield the lowest value (0.24). Regarding the supporting measures, H2PC, HC, MMHC, RSMAX2, and Tabu achieve the lowest SHD (2.0), whereas several algorithms share the highest F1 value (0.65).

With the exception of IAMB-F, all remaining algorithms exhibit a high degree of similarity in their performance values. Notably, IAMB-F generates intermediate performance across most metrics. Consequently, according to the overall rank, retained as a complementary summary measure, IAMB-F emerges as the best-performing algorithm for DAG_3.

An examination of additional sample sizes reported in the Supplementary Document reveals that MCC values remain highly similar for several algorithms across sample sizes, although some changes in relative performance are observed. Unlike its behavior in DAG_1 and DAG_2, the PCS algorithm—which generally exhibits improving metric values as the sample size increases—shows little change in DAG_3 according to the primary metric. Its MCC changes only from 0.49 at *n* = 200 to 0.50 at *n* = 500, 1000, and 2500.

**Table 6:** Selected performance metrics and overall ranking of BN learning algorithms for DAG_3 with sample size *n* = 200.

	MCC	R_MCC_	SHD	R_SHD_	F1	R_F1_	Overall Rank
**F-IAMB**	0.50 (0.02)	4	2.2 (0.56)	10.5	0.65 (0.05)	4.5	7.5
**GS**	0.50 (0.02)	4	2.2 (0.56)	10.5	0.65 (0.05)	4.5	7.5
**HPC**	0.50 (0.02)	4	2.2 (0.56)	10.5	0.65 (0.05)	4.5	7.5
**IAMB**	0.50 (0.02)	4	2.2 (0.56)	10.5	0.65 (0.05)	4.5	7.5
**I-IAMB**	0.50 (0.02)	4	2.2 (0.56)	10.5	0.65 (0.05)	4.5	7.5
**MMPC**	0.50 (0.02)	4	2.2 (0.56)	10.5	0.65 (0.05)	4.5	7.5
**SPC**	0.50 (0.02)	4	2.2 (0.56)	10.5	0.65 (0.05)	4.5	7.5
**IAMB-F**	0.49 (0.05)	8	2.1 (0.44)	6	0.65 (0.05)	4.5	5.7
**PCS**	0.49 (0.06)	9	2.2 (0.56)	10.5	0.64 (0.06)	9	9.3
**H2PC**	0.24 (0.05)	12	2.0 (0.18)	3	0.50 (0.02)	12	7.5
**HC**	0.24 (0.05)	12	2.0 (0.18)	3	0.50 (0.02)	12	7.5
**MMHC**	0.24 (0.05)	12	2.0 (0.18)	3	0.50 (0.02)	12	7.5
**RSMAX2**	0.24 (0.05)	12	2.0 (0.18)	3	0.50 (0.02)	12	7.5
**Tabu**	0.24 (0.05)	12	2.0 (0.18)	3	0.50 (0.02)	12	7.5

Values in parentheses indicate standard deviations. F-IAMB: Fast-IAMB; GS: Grow-Shrink; H2PC: Hybrid HPC; HC: Hill-Climbing; HPC: Hybrid Parents and Children; IAMB: Incremental association Markov Blanket; IAMB-F: IAMB with False Discovery Rate Correction; I-IAMB: Interleaved IAMB; MMHC: Max–Min Hill-Climbing; MMPC: Max–Min Parents and Children; PCS: Peter–Clark Stable; RSMAX2: Restricted Maximization; SPC: Semi-Interleaved HITON-PC; Tabu: Tabu search. MCC: Matthews Correlation Coefficient; SHD: Directed Structural Hamming Distance; F1: F1 Score; R: Rank; Overall Rank is based on the rankings across all evaluated performance metrics; the complete metric-specific results are provided in Appendix A.

The results for DAG_4 with *n* = 200 observations are reported in Table 7. According to MCC, IAMB-F achieves the highest ranking with a mean value of 0.55, although several other algorithms show the same rounded mean value. Regarding the supporting measures, PCS achieves the lowest SHD (4.5), whereas IAMB-F produces the highest F1 score (0.66). IAMB-F ranks first according to the overall ranking, followed by PCS as the second-best algorithm. As observed in DAG_1 and DAG_2, HC consistently ranks last.

When examining other sample sizes in the Supplementary Document for DAG_4, the MCC results show that HPC, MMPC, and SPC become the strongest-performing algorithms as *n* increases. Their MCC values are 0.54 at *n* = 500, 0.52 at *n* = 1000, and 0.46 at *n* = 2500. In comparison, the MCC of IAMB-F declines from 0.55 at *n* = 200 to 0.44 at *n* = 2500, while PCS decreases from 0.53 to 0.41. HC, Tabu, and the hybrid algorithms consistently produce the weakest MCC values across larger sample sizes.

Overall, DAG_4 highlights a strong separation between the stronger-performing constraint-based methods and the weaker hybrid algorithms, particularly according to MCC at larger sample sizes. For DAG_4, MCC values generally decrease as the sample size increases, although the magnitude of this decline differs considerably among algorithms.

**Table 7:** Selected performance metrics and overall ranking of BN learning algorithms for DAG_4 with sample size *n* = 200.

	MCC	R_MCC_	SHD	R_SHD_	F1	R_F1_	Overall Rank
**IAMB-F**	0.55 (0.07)	1	4.8 (0.84)	2	0.66 (0.05)	1	2.5
**HPC**	0.55 (0.05)	2	5.3 (0.73)	6	0.65 (0.03)	3	5.3
**MMPC**	0.55 (0.05)	3	5.3 (0.77)	8	0.65 (0.03)	4	6.6
**SPC**	0.55 (0.05)	4	5.3 (0.77)	9	0.65 (0.03)	5	7.4
**IAMB**	0.55 (0.06)	5.5	5.3 (0.90)	3.5	0.65 (0.04)	6.5	5.1
**I-IAMB**	0.55 (0.06)	5.5	5.3 (0.90)	3.5	0.65 (0.04)	6.5	5.1
**GS**	0.55 (0.06)	7	5.3 (0.90)	5	0.65 (0.04)	8	6.6
**F-IAMB**	0.55 (0.06)	8	5.3 (0.89)	7	0.65 (0.04)	9	7.6
**PCS**	0.53 (0.14)	9	4.5 (1.5)	1	0.65 (0.10)	2	3.1
**Tabu**	−0.23 (0.22)	10	9.3 (1.6)	10	0.30 (0.18)	10	9.1
**H2PC**	−0.26 (0.15)	11	9.5 (1.1)	11	0.24 (0.12)	11	10.2
**MMHC**	−0.26 (0.15)	12	9.5 (1.1)	12	0.23 (0.11)	12.5	11.4
**RSMAX2**	−0.26 (0.15)	13	9.5 (1.1)	13	0.23 (0.11)	12.5	12.1
**HC**	−0.27 (0.15)	14	9.6 (1.2)	14	0.23 (0.11)	14	12.8

Values in parentheses indicate standard deviations. F-IAMB: Fast-IAMB; GS: Grow-Shrink; H2PC: Hybrid HPC; HC: Hill-Climbing; HPC: Hybrid Parents and Children; IAMB: Incremental association Markov Blanket; IAMB-F: IAMB with False Discovery Rate Correction; I-IAMB: Interleaved IAMB; MMHC: Max–Min Hill-Climbing; MMPC: Max–Min Parents and Children; PCS: Peter–Clark Stable; RSMAX2: Restricted Maximization; SPC: Semi-Interleaved HITON-PC; Tabu: Tabu search. MCC: Matthews Correlation Coefficient; SHD: Directed Structural Hamming Distance; F1: F1 Score; R: Rank; Overall Rank is based on the rankings across all evaluated performance metrics; the complete metric-specific results are provided in Appendix A.

The results for DAG_5 with *n* = 200 observations are presented in Table 8. For DAG_5, HPC produces the best performance according to the primary metric, MCC, and also ranks first according to the supporting F1 measure. IAMB-F achieves the best SHD and ranks first overall.

Results for other sample sizes are reported in the Supplementary Document. According to the primary metric, PCS shows the clearest improvement as the sample size increases, with its MCC rising from 0.38 at *n* = 200 to 0.47 at *n* = 500 and 0.54 at *n* = 1000, before declining to 0.42 at *n* = 2500. In contrast, HPC declines from 0.49 at *n* = 200 to 0.35 at *n* = 2500, while HC, Tabu, and the hybrid algorithms remain among the weakest performers according to MCC. Overall, most algorithms exhibit lower MCC values at the largest sample size, whereas PCS retains a higher MCC at *n* = 2500 than at *n* = 200.

**Table 8:** Selected performance metrics and overall ranking of BN learning algorithms for DAG_5 with sample size *n* = 200.

	MCC	R_MCC_	SHD	R_SHD_	F1	R_F1_	Overall Rank
**HPC**	0.49 (0.07)	1	6.4 (0.79)	7	0.64 (0.03)	1	5
**SPC**	0.49 (0.07)	2	6.5 (0.87)	8	0.64 (0.04)	2	5.8
**MMPC**	0.49 (0.07)	3	6.5 (0.88)	9	0.64 (0.04)	3	6.6
**GS**	0.48 (0.14)	4	6.3 (1.5)	3	0.63 (0.09)	4	4.5
**IAMB**	0.48 (0.14)	5.5	6.3 (1.4)	4.5	0.63 (0.09)	6.5	5.9
**I-IAMB**	0.48 (0.14)	5.5	6.3 (1.4)	4.5	0.63 (0.09)	6.5	5.9
**IAMB-F**	0.48 (0.20)	7	5.7 (1.9)	1	0.63 (0.14)	5	3.9
**F-IAMB**	0.48 (0.14)	8	6.4 (1.4)	6	0.63 (0.09)	8	7.1
**PCS**	0.38 (0.18)	9	6.0 (1.5)	2	0.58 (0.11)	9	6
**Tabu**	0.17 (0.30)	10	7.1 (2.6)	10	0.43 (0.20)	10	8.9
**RSMAX2**	0.10 (0.26)	11	7.6 (2.2)	11	0.38 (0.17)	13	10.5
**MMHC**	0.10 (0.26)	12	7.6 (2.2)	12	0.38 (0.17)	12	11
**H2PC**	0.09 (0.26)	13	7.6 (2.1)	13	0.38 (0.17)	14	11.9
**HC**	0.09 (0.27)	14	7.7 (2.4)	14	0.38 (0.18)	11	12.1

Values in parentheses indicate standard deviations. F-IAMB: Fast-IAMB; GS: Grow-Shrink; H2PC: Hybrid HPC; HC: Hill-Climbing; HPC: Hybrid Parents and Children; IAMB: Incremental association Markov Blanket; IAMB-F: IAMB with False Discovery Rate Correction; I-IAMB: Interleaved IAMB; MMHC: Max–Min Hill-Climbing; MMPC: Max–Min Parents and Children; PCS: Peter–Clark Stable; RSMAX2: Restricted Maximization; SPC: Semi-Interleaved HITON-PC; Tabu: Tabu search. MCC: Matthews Correlation Coefficient; SHD: Directed Structural Hamming Distance; F1: F1 Score; R: Rank; Overall Rank is based on the rankings across all evaluated performance metrics; the complete metric-specific results are provided in Appendix A.

**Figure 2:**
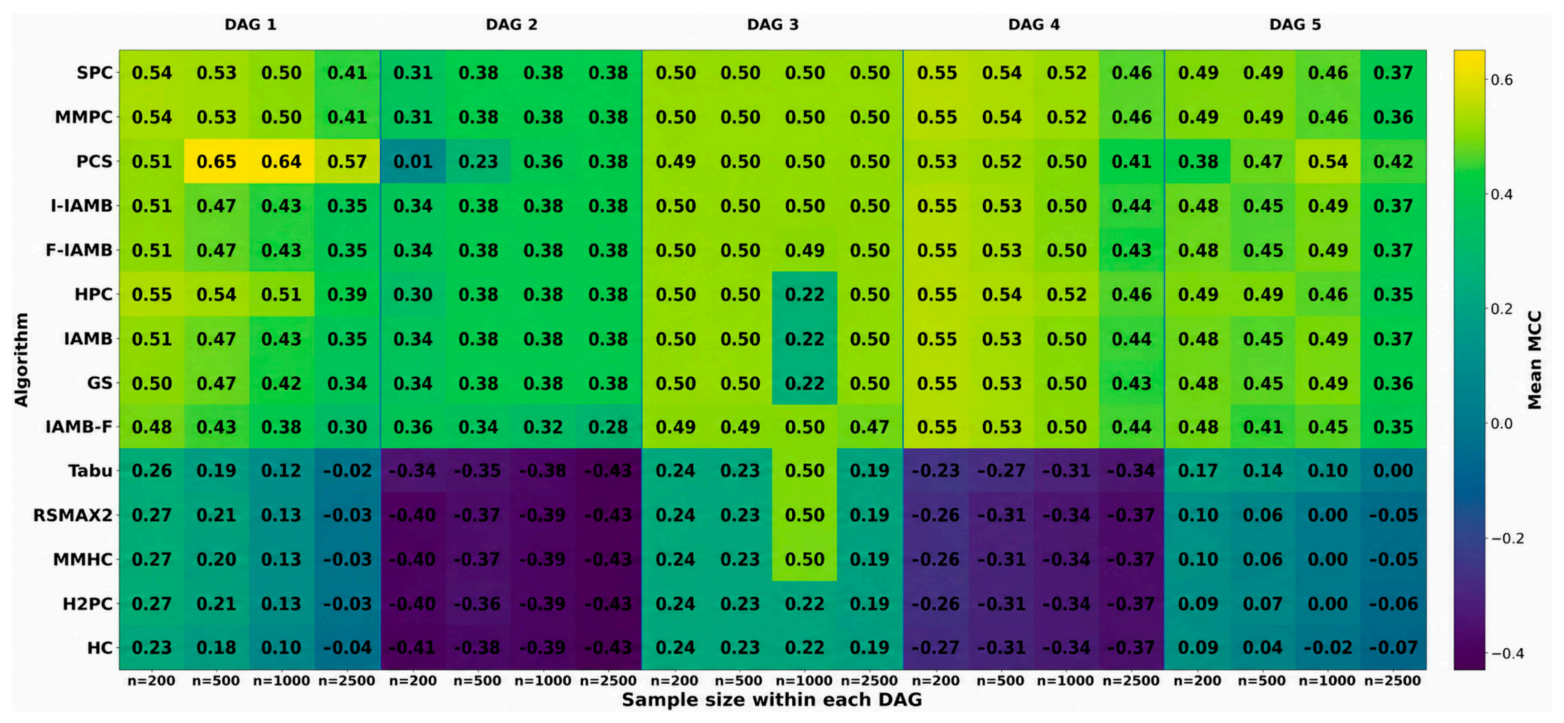
Mean MCC heatmap across DAG structures and sample sizes.

Figure 2 provides an overall comparison of algorithm performance based on the primary metric, MCC. Algorithms are ordered according to their mean MCC across all DAG structures and sample sizes, from the highest to the lowest average performance. The heatmap shows that algorithm performance varies considerably across DAG structures and sample sizes, indicating that no single algorithm consistently dominates under all simulation conditions. PCS shows particularly strong improvements with increasing sample size in some structures, especially DAG_1 and DAG_5, whereas HPC, MMPC, and SPC generally maintain relatively strong MCC performance across several DAGs. In contrast, HC, Tabu, and the hybrid algorithms tend to produce lower MCC values, particularly in DAG_2 and DAG_4.

As shown in Supplementary Table S21, hyperparameter tuning produced some condition-specific changes in performance; however, the overall comparative pattern across algorithms remained broadly consistent with the default-setting results. Computational efficiency also varied across algorithms and sample sizes. As shown in Supplementary Table S22, SPC had the lowest overall mean solution time (2.01 ms per dataset), whereas Tabu had the highest (9.59 ms per dataset). Mean solution times generally increased with sample size, although the absolute computational burden remained low under the relatively small DAG structures examined. A cross-software sensitivity analysis using the PC algorithm showed only small differences between the bnlearn and TETRAD implementations, with overall MCC values of 0.456 and 0.445, respectively; F1 and SHD showed similarly small differences (Supplementary Table S23).

## 5. Discussion

This study investigates the performance of Bayesian Network (BN) algorithms under a comprehensive simulation framework involving multiple sample sizes and randomly generated directed acyclic graphs (DAGs) with latent constructs. By evaluating 14 algorithms across different graph topologies and observation levels, the study provides nuanced assessment of algorithmic behavior that goes beyond conclusions drawn from single-structure or single-metric evaluations. Unlike earlier studies relying on a fixed model or single metric, the present design allows performance patterns to be examined across structurally diverse settings, thereby offering a more generalizable perspective on causal discovery performance.

A central finding of the study is that algorithm performance is highly sensitive to the underlying DAG structure [28]. While certain algorithms perform well under specific graph configurations, their relative rankings may change substantially as the structural complexity, edge orientation, or dependency patterns vary. This observation is particularly evident for Matthews correlation coefficient (MCC), which was used as the primary performance metric, and is further supported by F1 and directed structural Hamming distance (SHD). Consequently, no single algorithm uniformly dominates across all DAGs and metrics [24], highlighting the importance of considering graph-specific characteristics when selecting BN learning methods.

Contrary to common expectations in the literature [15,16], hybrid algorithms (Max–Min Hill-Climbing, Hybrid HPC, and Restricted Maximization) did not consistently outperform constraint-based or score-based algorithms in this study. In particular, across multiple DAGs and sample sizes, hybrid algorithms exhibited generally weak or unstable performance according to MCC and the supporting F1 score and SHD measures, and their overall rankings were often inferior to those of simpler constraint-based approaches. One possible explanation is that the two-stage nature of hybrid methods may propagate errors from the constraint-based identification of candidate relationships to the subsequent score-based search, particularly when dependency patterns vary across DAG structures. This finding challenges the prevailing assumption that hybrid algorithms universally provide superior causal discovery performance, and suggests that their effectiveness may depend more critically on structural and distributional assumptions than previously recognized.

Constraint-based algorithms displayed heterogeneous but in some cases remarkably robust behavior [30,31]. Algorithms such as Peter–Clark Stable, Hybrid Parents and Children, and Max–Min Parents and Children demonstrated strong and interpretable performance patterns that varied systematically with graph structure and sample size. In particular, Peter–Clark Stable exhibited an unusual but consistent increase in MCC for certain DAGs as the sample size grew, in contrast to the general tendency of other algorithms to experience performance degradation with larger *n*. This pattern was particularly evident in DAG_1, DAG_2, and DAG_5, suggesting that Peter–Clark Stable may benefit from increased statistical power in specific dependency structures. Because PCS relies on conditional independence tests, larger samples may improve the statistical power of these tests and reduce erroneous edge decisions [24]. However, this benefit may differ across DAG structures because the performance of conditional independence tests depends on the underlying dependency patterns and conditioning sets [24]. Therefore, larger sample sizes do not necessarily lead to similar performance improvements across all DAGs.

Conversely, algorithms such as Hill-Climbing showed generally weak MCC values and overall rankings across the examined DAGs, indicating limited robustness to structural variability.

Another considerable finding is the presence of metric saturation effects in simpler DAG structures. For DAGs with a smaller number of latent variables and relations (e.g., DAG_3), many algorithms produced nearly identical metric values, including MCC and F1. This phenomenon suggests that when the causal structure is relatively simple, algorithmic differences may be masked, and performance rankings become less informative. In such settings, SHD provides useful complementary structural information alongside MCC, reinforcing the value of evaluating algorithm performance using more than one criterion.

From a methodological standpoint, the study demonstrates that evaluating BN algorithms using multiple DAG structures and performance metrics yields a more realistic and cautious interpretation of algorithmic superiority. The results indicate that algorithm rankings derived from a single structure or aggregate metric may be misleading, as performance advantages often fail to generalize across alternative structural configurations. Accordingly, the main results emphasize MCC as the primary metric while retaining SHD and F1 as complementary measures of structural recovery and balanced performance.

## 6. Conclusions

This study provides a comprehensive evaluation of BN learning algorithms under a simulation framework that jointly considers multiple sample sizes and randomly generated DAGs with latent constructs. By analyzing algorithm performance across diverse structural configurations rather than a single model, the findings demonstrate that algorithm effectiveness depends not only on sample size but, more critically, on the underlying causal structure of the data-generating process.

One of the main contributions of this study is addressing a notable gap in the literature regarding the lack of systematic, structure-diverse, and systematically validated comparisons of BN learning algorithms. While many previous studies rely on limited structural settings or focus on a narrow subset of performance metrics, this study evaluates 14 widely used algorithms across multiple DAGs and a broad set of both classification-based and structure-aware metrics, including directed structural Hamming distance. For the main comparative assessment, MCC was designated as the primary metric, with SHD and F1 serving as supporting measures. The use of SEM-based validation further ensures that the simulated datasets are structurally coherent and methodologically sound.

The results reveal that no single class of algorithms consistently dominates across all metrics and DAG structures. In contrast to common assumptions in the literature, hybrid algorithms did not universally outperform constraint-based or score-based methods. Although hybrid approaches are often designed to balance statistical efficiency and search optimality, their performance in this study was frequently unstable and, in several scenarios, inferior to simpler constraint-based algorithms. These findings suggest that the advantages of hybrid methods are more context-dependent than previously assumed and may diminish under certain structural or sample size conditions.

Constraint-based algorithms exhibited heterogeneous yet, in some cases, remarkably stable behavior. Algorithms such as Peter–Clark Stable, Hybrid Parents and Children, and Max–Min Parents and Children showed strong performance in specific DAG configurations, with Peter–Clark Stable in particular displaying an atypical improvement in MCC as sample size increased for certain structures. At the same time, consistently weak performance observed for algorithms such as Hill-Climbing across almost all scenarios highlights that scalability and structural adaptability remain critical challenges in causal discovery, while scalability requires further evaluation using larger DAG structures.

From a practical perspective, these results underscore the importance of aligning algorithm choice with data characteristics rather than relying on algorithm class labels alone. Structural complexity, edge orientation, and metric priorities (e.g., MCC-based balanced edge recovery versus SHD-based structural recovery) play a decisive role in determining algorithm suitability. Within the conditions examined, PCS may be preferred when moderate-to-large samples are available and both balanced edge recovery and structural recovery are priorities, whereas IAMB-F may be considered for simpler structures when classification-oriented performance is emphasized. When exact structural recovery is the primary objective, SHD could be considered explicitly, particularly when classification-based metrics yield similar results. Consequently, practitioners are encouraged to adopt a metric-aware and structure-aware approach when selecting causal discovery tools.

Although the present study relies on a simulation framework, the use of randomly generated DAGs and varying sample sizes mirrors realistic causal discovery settings in which the true structure is unknown and must be inferred from data. As such, the simulation design enhances external validity by approximating real-world uncertainty rather than representing a purely artificial evaluation setup.

This study examines a selected set of widely used constraint-based, score-based, and hybrid Bayesian network learning algorithms under partially controlled simulation setting. Other causal discovery families, such as Greedy Equivalence Search and its variants, Fast Causal Inference for settings with latent confounding, and optimization-based approaches such as Nonlinear Optimization via Trace Exponential and Augmented lagRangian for Structure learning, were not included in the simulation-based evaluation. These methods rely on substantially different modeling assumptions, estimation targets, or optimization frameworks, which would require different simulation structures and evaluation criteria to ensure fair comparison. A systematic application of such approaches under appropriately aligned simulation structures therefore represents a natural and important direction for future research.

Despite its contributions, the study has some limitations. Although multiple random DAGs were considered, the number of latent variables was intentionally restricted to maintain interpretability and computational feasibility. Future research could explore larger and more densely connected DAGs to assess scalability effects more thoroughly. Computational performance could also be examined under big-data settings, where the curse of dimensionality may become more pronounced. In addition, averaging the three indicators of each latent construct into a single manifest score simplified the input to the BN algorithms but may have masked indicator-specific variation and measurement-level relationships; future studies could compare aggregated and indicator-level representations. All algorithms were evaluated using their default parameter settings in the primary analysis, reflecting common out-of-the-box usage and providing a standardized baseline for comparison. An additional algorithm-specific hyperparameter tuning analysis was conducted as a sensitivity analysis. Although tuning produced some condition-specific changes, the overall comparative pattern remained broadly consistent with the default-setting results; broader hyperparameter spaces and alternative tuning strategies could be examined in future studies. All algorithms were implemented within a single R/bnlearn environment to maintain a consistent software framework. A cross-software sensitivity analysis using TETRAD revealed small implementation-specific differences, although the overall performance pattern remained comparable. Nevertheless, broader cross-platform replication involving additional algorithms and parameter settings may provide further insight into software-specific variability.

## Figures and Tables

**Table 1:** Comparison of selected related studies on Bayesian network and causal structure learning.

Study	Year	Main Scope	Data/Structural Setting	Experimental Variation	Evaluation
Singh, Gupta, Tewari and Shroff [18]	2017	Comparative benchmarking of causal discovery techniques	Two real datasets and one simulated dataset	Small, medium, and large sample size settings	AUC, F-score, SHD, SID, predictive and counterfactual accuracy
Farnia, Alibegovic and Cruickshank [19]	2023	Joint assessment and ranking of causal structure learning algorithms	Linear data-generating settings with latent-confounding scenarios	Medium and large samples; different latent-confounding conditions	Efficiency and effectiveness
Constantinou, Liu, Chobtham, Guo and Kitson [23]	2021	Large-scale comparison of 15 BN structure learning algorithms	Synthetic networks of varying complexity with clean and noisy data	Network complexity, sample size, and multiple types/levels of noise	F1, SHD, BSF; also computational performance and robustness
Kitson and Constantinou [25]	2024	Sensitivity of BN structure learning to variable ordering	Discrete categorical data and multiple underlying networks	Variable ordering, sample size, objective score, and hyperparameters	Accuracy of learned graph/CPDAG
Velev and Lessmann [26]	2025	Multidimensional comparison of 14 causal discovery techniques	Simulated nonlinear, non-identifiable causal settings	Seven experimental factors; 215,040 model runs	Multidimensional DOS and EBM framework
Chobtham and Constantinou [29]	2024	Hyperparameter tuning for structure learning	Synthetic experiments and case studies	Algorithms, sample sizes, dimensionality, and hyperparameter settings	F1 and SHD; score-based tuning criteria
Yu, Ling, Liu, Li, Wang and Li [30]	2023	Efficient local-to-global BN structure learning	Six benchmark BNs	Comparison with eight existing BN learning algorithms	Computational efficiency and structure learning quality
Wang, Liu, Zhang, Cai and Shi [32]	2024	Hybrid BN structure learning using efficient skeleton learning	Multiple benchmark BNs	Evaluation across 11 BNs for skeleton learning and nine BNs for structure learning	Structural accuracy and computational efficiency
Göbler, Windisch, Drton, Pychynski, Roth and Sonntag [33]	2024	Generation of realistic benchmark data for causal discovery	Semisynthetic production data with known ground-truth causal relationships	Controlled generation of benchmark datasets	Benchmarking of several causal discovery algorithms

AUC: Area Under the Curve; SID: Structural Intervention Distance; BSF: Balanced Scoring Function; CPDAG: Completed Partially Directed Acyclic Graph; DOS: Distance to the Optimal Solution; EBM: Explainable Boosting Machine.

**Table 2:** Confusion Matrix.

		Actual Values	
		+	−
Predicted Values	+	True Positive (TP)	False Positive (FP)
	−	False Negative (FN)	True Negative (TN)

**Table 3:** Formulas of performance metrics.

Performance Metric	Abbreviation	Formula
Accuracy	ACC	$\frac{TP+TN}{TP+FP+FN+TN}$
Sensitivity (Recall)	SEN	$\frac{TP}{TP+FN}$
Precision	PRE	$\frac{TP}{TP+FP}$
Specificity	SPE	$\frac{TN}{FP+TN}$
F1 Score	F1	$\frac{2\times (PRE\times SEN)}{\left(PRE+SEN\right)}$
Cohen’s kappa	CK	$\frac{ACC-P_{c}}{1-P_{c}}$ $P_{c}=\frac{\left(TP+FN\right)\times \left(TP+FP\right)+\left(TN+FN\right)\times (TN+FP)}{{\left(TP+FP+FN+TN\right)}^{2}}$
Matthews correlation coefficient	MCC	$\frac{\left(TP\times TN)-(FP\times FN\right)}{\sqrt{\left(TP+FP\right)\times \left(TN+FN\right)\times \left(TP+FN\right)\times \left(TN+FP\right)}}$
Directed Structural Hamming Distance	SHD	*EE* + *ME*

Note: *TP*, *TN*, *FP*, and *FN* denote true positives, true negatives, false positives, and false negatives, respectively; $P_{c}$ denotes the probability of chance agreement in Cohen’s kappa; *EE* denotes extra edges and *ME* missing edges with respect to the original structure. Under the directed edge-wise comparison used in this study, a reversed edge contributes one missing edge and one extra edge.

## Data Availability

All data were generated via Monte Carlo simulations. The R scripts for data generation and for implementing all BN learning algorithms are publicly available at https://github.com/tugaykaradag/semsimbn_2026 (accessed on 23 August 2026). Although replication-level seeds were not stored, the simulation design, parameter settings, and software versions are fully documented, ensuring reproducibility at the distributional level.

## Supplementary Materials

The following supporting information can be downloaded at https://www.mdpi.com/article/10.3390/e28090955/s1: Table S1: Descriptive statistics of Goodness-of-Fit measures for DAG_1; Table S2: Descriptive statistics of Goodness-of-Fit measures for DAG_2; Table S3: Descriptive statistics of Goodness-of-Fit measures for DAG_3; Table S4: Descriptive statistics of Goodness-of-Fit measures for DAG_4; Table S5: Descriptive statistics of Goodness-of-Fit measures for DAG_5; Table S6: Algorithm performance for DAG_1 with sample size *n* = 500; Table S7: Algorithm performance for DAG_1 with sample size *n* = 1000; Table S8: Algorithm performance for DAG_1 with sample size *n* = 2500; Table S9: Algorithm performance for DAG_2 with sample size *n* = 500; Table S10: Algorithm performance for DAG_2 with sample size *n* = 1000; Table S11: Algorithm performance for DAG_2 with sample size *n* = 2500; Table S12: Algorithm performance for DAG_3 with sample size *n* = 500; Table S13: Algorithm performance for DAG_3 with sample size *n* = 1000; Table S14: Algorithm performance for DAG_3 with sample size *n* = 2500; Table S15: Algorithm performance for DAG_4 with sample size *n* = 500; Table S16: Algorithm performance for DAG_4 with sample size *n* = 1000; Table S17: Algorithm performance for DAG_4 with sample size *n* = 2500; Table S18: Algorithm performance for DAG_5 with sample size *n* = 500; Table S19: Algorithm performance for DAG_5 with sample size *n* = 1000; Table S20: Algorithm performance for DAG_5 with sample size *n* = 2500; Table S21: Mean changes in MCC, F1, and SHD after hyperparameter tuning across all simulation conditions; Table S22: Mean solution time (ms per dataset) of the Bayesian network learning algorithms across sample sizes; Table S23: Cross-software comparison of PC performance between bnlearn and TETRAD.

## Abbreviations

The following abbreviations are used in this manuscript:

ACC	Accuracy
AGFI	Adjusted Goodness-of-Fit Index
BN	Bayesian Network
BN-LV	Bayesian Networks for Latent Variables
CFI	Comparative Fit Index
CK	Cohen’s Kappa
DAG	Directed Acyclic Graph
EE	Extra Edges
F1	F1 Score
FAS	Fast Adjacency Search
FN	False Negative
FP	False Positive
GFI	Goodness-of-Fit Index
GS	Grow-Shrink
H2PC	Hybrid HPC
HC	Hill-Climbing
HPC	Hybrid Parents and Children
IAMB	Incremental Association Markov Blanket
IAMB-F	IAMB with False Discovery Rate Correction
IFI	Incremental Fit Index
Inter-IAMB	Interleaved IAMB
MCC	Matthews Correlation Coefficient
ME	Missing Edges
MMHC	Max–Min Hill-Climbing
MMPC	Max–Min Parents and Children
NFI	Normed Fit Index
NNFI	Non-Normed Fit Index
PC	Peter–Clark
PCS	Peter–Clark Stable
PRE	Precision
RMSEA	Root Mean Square Error of Approximation
RSMAX2	Restricted Maximization
SEM	Structural Equation Modeling
SEN	Sensitivity
SHD	Structural Hamming Distance
SI-HITON-PC	Semi-Interleaved HITON Parents and Children
SPC	Semi-Interleaved HITON-PC
SPE	Specificity
SRMR	Standardized Root Mean Squared Residual
TN	True Negative
TP	True Positive

## Appendix A

This appendix presents the complete metric-specific performance results for the five DAGs at *n* = 200. The main text reports the primary and supporting performance measures, whereas the tables below provide the full set of evaluated metrics.

**Table A1:** Algorithm performance for DAG_1 with sample size *n* = 200.

	ACC	R_ACC_	SEN	R_SEN_	PRE	R_PRE_	SPE	R_SPE_	F1	R_F1_	CK	R_CK_	MCC	R_MCC_	SHD	R_SHD_	Overall Rank
**PCS**	0.78 (0.09)	1	0.79 (0.17)	9	0.55 (0.13)	1	0.78 (0.08)	6	0.65 (0.15)	2	0.49 (0.21)	1	0.51 (0.21)	4	4.4 (1.9)	1	3.1
**HPC**	0.73 (0.04)	3	0.98 (0.06)	1	0.48 (0.04)	2	0.65 (0.05)	12	0.65 (0.04)	1	0.47 (0.06)	2	0.55 (0.06)	1	5.4 (0.82)	3	3.1
**MMPC**	0.73 (0.05)	5	0.98 (0.06)	2	0.48 (0.04)	3.5	0.64 (0.06)	14	0.64 (0.04)	3	0.46 (0.07)	3	0.54 (0.07)	2	5.5 (0.91)	5	4.7
**SPC**	0.73 (0.05)	4	0.98 (0.06)	3	0.48 (0.04)	3.5	0.64 (0.06)	13	0.64 (0.04)	4	0.46 (0.07)	4	0.54 (0.07)	3	5.5 (0.91)	4	4.8
**IAMB-F**	0.73 (0.04)	2	0.86 (0.22)	8	0.48 (0.06)	5	0.69 (0.07)	7	0.61 (0.10)	9	0.43 (0.12)	9	0.48 (0.14)	9	5.3 (0.80)	2	6.4
**IAMB**	0.72 (0.05)	10.5	0.92 (0.18)	5.5	0.47 (0.06)	6.5	0.66 (0.07)	9.5	0.62 (0.08)	6.5	0.44 (0.11)	6.5	0.51 (0.13)	6.5	5.6 (0.99)	10.5	7.8
**I-IAMB**	0.72 (0.05)	10.5	0.92 (0.18)	5.5	0.47 (0.06)	6.5	0.66 (0.07)	9.5	0.62 (0.08)	6.5	0.44 (0.11)	6.5	0.51 (0.13)	6.5	5.6 (0.99)	10.5	7.8
**F-IAMB**	0.72 (0.05)	12	0.93 (0.17)	4	0.47 (0.06)	9	0.65 (0.07)	11	0.62 (0.08)	5	0.44 (0.11)	5	0.51 (0.12)	5	5.6 (0.99)	12	7.9
**GS**	0.72 (0.05)	8	0.91 (0.18)	7	0.47 (0.06)	8	0.66 (0.08)	8	0.62 (0.09)	8	0.43 (0.11)	8	0.50 (0.13)	8	5.5 (0.98)	8	7.9
**H2PC**	0.72 (0.15)	6	0.46 (0.30)	10	0.45 (0.30)	10	0.81 (0.11)	1	0.50 (0.28)	11	0.27 (0.41)	10	0.27 (0.41)	10	5.5 (3.1)	6	8.0
**MMHC**	0.72 (0.15)	7	0.46 (0.30)	11	0.45 (0.30)	11	0.81 (0.11)	3	0.50 (0.28)	12	0.27 (0.41)	11	0.27 (0.41)	11	5.5 (3.1)	7	9.1
**RSMAX2**	0.72 (0.15)	9	0.45 (0.31)	13	0.45 (0.31)	12	0.81 (0.11)	2	0.50 (0.28)	10	0.27 (0.41)	12	0.27 (0.41)	12	5.5 (3.1)	9	9.9
**Tabu**	0.72 (0.15)	13	0.46 (0.28)	12	0.44 (0.28)	13	0.80 (0.10)	4	0.49 (0.25)	13	0.26 (0.37)	13	0.26 (0.38)	13	5.7 (2.9)	13	11.8
**HC**	0.70 (0.16)	14	0.44 (0.30)	14	0.42 (0.30)	14	0.79 (0.11)	5	0.48 (0.28)	14	0.23 (0.41)	14	0.23 (0.41)	14	5.9 (3.2)	14	12.9

Values in parentheses indicate standard deviations. F-IAMB: Fast-IAMB; GS: Grow-Shrink; H2PC: Hybrid HPC; HC: Hill-Climbing; HPC: Hybrid Parents and Children; IAMB: Incremental association Markov Blanket; IAMB-F: IAMB with False Discovery Rate Correction; I-IAMB: Interleaved IAMB; MMHC: Max–Min Hill-Climbing; MMPC: Max–Min Parents and Children; PCS: Peter–Clark Stable; RSMAX2: Restricted Maximization; SPC: Semi-Interleaved HITON-PC; Tabu: Tabu search. ACC: Accuracy; SEN: Sensitivity; PRE: Precision; SPE: Specificity; F1: F1 Score; CK: Cohen’s Kappa; MCC: Matthews Correlation Coefficient; SHD: Directed Structural Hamming Distance; R: Rank.

**Table A2:** Algorithm performance for DAG_2 with sample size *n* = 200.

	ACC	R_ACC_	SEN	R_SEN_	PRE	R_PRE_	SPE	R_SPE_	F1	R_F1_	CK	R_CK_	MCC	R_MCC_	SHD	R_SHD_	Overall Rank
**IAMB-F**	0.61 (0.12)	1	0.85 (0.19)	8	0.55 (0.16)	1	0.44 (0.26)	5	0.65 (0.11)	1	0.28 (0.21)	1	0.36 (0.21)	1	4.6 (1.4)	1	2.4
**IAMB**	0.58 (0.09)	3	0.92 (0.14)	4.5	0.51 (0.11)	3	0.34 (0.19)	8.5	0.65 (0.08)	2.5	0.23 (0.16)	2.5	0.34 (0.17)	2.5	5.0 (1.1)	3	3.7
**I-IAMB**	0.58 (0.09)	3	0.92 (0.14)	4.5	0.51 (0.11)	3	0.34 (0.19)	8.5	0.65 (0.08)	2.5	0.23 (0.16)	2.5	0.34 (0.17)	2.5	5.0 (1.1)	3	3.7
**GS**	0.58 (0.09)	3	0.92 (0.14)	6	0.51 (0.11)	3	0.34 (0.19)	7	0.65 (0.08)	5	0.23 (0.16)	4	0.34 (0.17)	4	5.0 (1.1)	3	4.4
**F-IAMB**	0.58 (0.09)	5	0.92 (0.13)	3	0.51 (0.11)	5	0.34 (0.19)	10	0.65 (0.08)	4	0.23 (0.16)	5	0.34 (0.17)	5	5.0 (1.1)	5	5.3
**MMPC**	0.56 (0.05)	7.5	0.94 (0.10)	1	0.49 (0.03)	7.5	0.30 (0.11)	14	0.64 (0.04)	6	0.21 (0.09)	7	0.31 (0.12)	6	5.2 (0.64)	7.5	7.1
**HPC**	0.57 (0.05)	6	0.92 (0.10)	7	0.49 (0.03)	6	0.32 (0.10)	11	0.64 (0.04)	8	0.21 (0.08)	6	0.30 (0.12)	8	5.2 (0.58)	6	7.3
**SPC**	0.56 (0.05)	7.5	0.94 (0.10)	2	0.49 (0.03)	7.5	0.30 (0.11)	13	0.64 (0.04)	7	0.21 (0.09)	8	0.31 (0.12)	7	5.2 (0.64)	7.5	7.4
**Tabu**	0.35 (0.15)	10	0.20 (0.19)	10	0.20 (0.19)	10	0.46 (0.13)	1	0.30 (0.16)	10	−0.34 (0.31)	10	−0.34 (0.31)	10	7.8 (1.8)	10	8.9
**PCS**	0.47 (0.13)	9	0.67 (0.26)	9	0.40 (0.12)	9	0.32 (0.13)	12	0.50 (0.15)	9	−0.01 (0.26)	9	0.01 (0.31)	9	6.4 (1.5)	9	9.4
**MMHC**	0.33 (0.12)	12	0.16 (0.16)	12	0.17 (0.17)	11	0.45 (0.10)	3.5	0.27 (0.12)	13	−0.40 (0.25)	11	−0.40 (0.25)	11	8.1 (1.4)	12	10.7
**H2PC**	0.33 (0.12)	11	0.16 (0.16)	14	0.16 (0.17)	14	0.45 (0.10)	2	0.27 (0.12)	11	−0.40 (0.25)	12	−0.40 (0.25)	13	8.1 (1.4)	11	11.0
**RSMAX2**	0.33 (0.12)	13	0.16 (0.16)	13	0.17 (0.17)	12	0.45 (0.10)	3.5	0.27 (0.12)	12	−0.40 (0.25)	13	−0.40 (0.25)	12	8.1 (1.4)	13	11.4
**HC**	0.32 (0.13)	14	0.16 (0.16)	11	0.16 (0.16)	13	0.43 (0.11)	6	0.27 (0.12)	14	−0.41 (0.26)	14	−0.41 (0.26)	14	8.2 (1.5)	14	12.5

Values in parentheses indicate standard deviations. F-IAMB: Fast-IAMB; GS: Grow-Shrink; H2PC: Hybrid HPC; HC: Hill-Climbing; HPC: Hybrid Parents and Children; IAMB: Incremental association Markov Blanket; IAMB-F: IAMB with False Discovery Rate Correction; I-IAMB: Interleaved IAMB; MMHC: Max–Min Hill-Climbing; MMPC: Max–Min Parents and Children; PCS: Peter–Clark Stable; RSMAX2: Restricted Maximization; SPC: Semi-Interleaved HITON-PC; Tabu: Tabu search. ACC: Accuracy; SEN: Sensitivity; PRE: Precision; SPE: Specificity; F1: F1 Score; CK: Cohen’s Kappa; MCC: Matthews Correlation Coefficient; SHD: Directed Structural Hamming Distance; R: Rank.

**Table A3:** Algorithm performance for DAG_3 with sample size *n* = 200.

	ACC	R_ACC_	SEN	R_SEN_	PRE	R_PRE_	SPE	R_SPE_	F1	R_F1_	CK	R_CK_	MCC	R_MCC_	SHD	R_SHD_	Overall Rank
**IAMB-F**	0.65 (0.07)	6	0.98 (0.09)	8	0.49 (0.04)	6	0.48 (0.12)	6	0.65 (0.05)	4.5	0.37 (0.09)	1	0.49 (0.05)	8	2.1 (0.44)	6	5.7
**H2PC**	0.66 (0.03)	3	0.50 (0.00)	12	0.49 (0.03)	3	0.74 (0.05)	3	0.50 (0.02)	12	0.24 (0.05)	12	0.24 (0.05)	12	2.0 (0.18)	3	7.5
**MMHC**	0.66 (0.03)	3	0.50 (0.00)	12	0.49 (0.03)	3	0.74 (0.05)	3	0.50 (0.02)	12	0.24 (0.05)	12	0.24 (0.05)	12	2.0 (0.18)	3	7.5
**RSMAX2**	0.66 (0.03)	3	0.50 (0.00)	12	0.49 (0.03)	3	0.74 (0.05)	3	0.50 (0.02)	12	0.24 (0.05)	12	0.24 (0.05)	12	2.0 (0.18)	3	7.5
**HC**	0.66 (0.03)	3	0.50 (0.00)	12	0.49 (0.03)	3	0.74 (0.05)	3	0.50 (0.02)	12	0.24 (0.05)	12	0.24 (0.05)	12	2.0 (0.18)	3	7.5
**Tabu**	0.66 (0.03)	3	0.50 (0.00)	12	0.49 (0.03)	3	0.74 (0.05)	3	0.50 (0.02)	12	0.24 (0.05)	12	0.24 (0.05)	12	2.0 (0.18)	3	7.5
**HPC**	0.64 (0.09)	10.5	1.0 (0.02)	4	0.49 (0.05)	10.5	0.46 (0.14)	11	0.65 (0.05)	4.5	0.37 (0.11)	5	0.50 (0.02)	4	2.2 (0.56)	10.5	7.5
**SPC**	0.64 (0.09)	10.5	1.0 (0.02)	4	0.49 (0.05)	10.5	0.46 (0.14)	11	0.65 (0.05)	4.5	0.37 (0.11)	5	0.50 (0.02)	4	2.2 (0.56)	10.5	7.5
**IAMB**	0.64 (0.09)	10.5	1.0 (0.02)	4	0.49 (0.05)	10.5	0.46 (0.14)	11	0.65 (0.05)	4.5	0.37 (0.11)	5	0.50 (0.02)	4	2.2 (0.56)	10.5	7.5
**I-IAMB**	0.64 (0.09)	10.5	1.0 (0.02)	4	0.49 (0.05)	10.5	0.46 (0.14)	11	0.65 (0.05)	4.5	0.37 (0.11)	5	0.50 (0.02)	4	2.2 (0.56)	10.5	7.5
**F-IAMB**	0.64 (0.09)	10.5	1.0 (0.02)	4	0.49 (0.05)	10.5	0.46 (0.14)	11	0.65 (0.05)	4.5	0.37 (0.11)	5	0.50 (0.02)	4	2.2 (0.56)	10.5	7.5
**MMPC**	0.64 (0.09)	10.5	1.0 (0.02)	4	0.49 (0.05)	10.5	0.46 (0.14)	11	0.65 (0.05)	4.5	0.37 (0.11)	5	0.50 (0.02)	4	2.2 (0.56)	10.5	7.5
**GS**	0.64 (0.09)	10.5	1.0 (0.02)	4	0.49 (0.05)	10.5	0.46 (0.14)	11	0.65 (0.05)	4.5	0.37 (0.11)	5	0.50 (0.02)	4	2.2 (0.56)	10.5	7.5
**PCS**	0.64 (0.09)	10.5	0.98 (0.11)	9	0.49 (0.05)	10.5	0.47 (0.15)	7	0.64 (0.06)	9	0.36 (0.11)	9	0.49 (0.06)	9	2.2 (0.56)	10.5	9.3

Values in parentheses indicate standard deviations. F-IAMB: Fast-IAMB; GS: Grow-Shrink; H2PC: Hybrid HPC; HC: Hill-Climbing; HPC: Hybrid Parents and Children; IAMB: Incremental association Markov Blanket; IAMB-F: IAMB with False Discovery Rate Correction; I-IAMB: Interleaved IAMB; MMHC: Max–Min Hill-Climbing; MMPC: Max–Min Parents and Children; PCS: Peter–Clark Stable; RSMAX2: Restricted Maximization; SPC: Semi-Interleaved HITON-PC; Tabu: Tabu search. ACC: Accuracy; SEN: Sensitivity; PRE: Precision; SPE: Specificity; F1: F1 Score; CK: Cohen’s Kappa; MCC: Matthews Correlation Coefficient; SHD: Directed Structural Hamming Distance; R: Rank.

**Table A4:** Algorithm performance for DAG_4 with sample size *n* = 200.

	ACC	R_ACC_	SEN	R_SEN_	PRE	R_PRE_	SPE	R_SPE_	F1	R_F1_	CK	R_CK_	MCC	R_MCC_	SHD	R_SHD_	Overall Rank
**IAMB-F**	0.76 (0.04)	2	0.92 (0.13)	8	0.52 (0.06)	2	0.70 (0.08)	2	0.66 (0.05)	1	0.50 (0.07)	2	0.55 (0.07)	1	4.8 (0.84)	2	2.5
**PCS**	0.78 (0.08)	1	0.83 (0.14)	9	0.55 (0.11)	1	0.76 (0.10)	1	0.65 (0.10)	2	0.50 (0.15)	1	0.53 (0.14)	9	4.5 (1.5)	1	3.1
**IAMB**	0.74 (0.05)	3.5	0.97 (0.08)	5.5	0.49 (0.05)	3.5	0.66 (0.07)	8.5	0.65 (0.04)	6.5	0.47 (0.06)	4.5	0.55 (0.06)	5.5	5.3 (0.90)	3.5	5.1
**I-IAMB**	0.74 (0.05)	3.5	0.97 (0.08)	5.5	0.49 (0.05)	3.5	0.66 (0.07)	8.5	0.65 (0.04)	6.5	0.47 (0.06)	4.5	0.55 (0.06)	5.5	5.3 (0.90)	3.5	5.1
**HPC**	0.73 (0.04)	6	0.98 (0.06)	3	0.49 (0.03)	7	0.65 (0.05)	12	0.65 (0.03)	3	0.47 (0.05)	3	0.55 (0.05)	2	5.3 (0.73)	6	5.3
**MMPC**	0.73 (0.04)	8	0.99 (0.05)	1.5	0.49 (0.03)	8	0.65 (0.05)	13	0.65 (0.03)	4	0.47 (0.06)	7	0.55 (0.05)	3	5.3 (0.77)	8	6.6
**GS**	0.74 (0.04)	5	0.97 (0.09)	7	0.49 (0.05)	5	0.66 (0.07)	10	0.65 (0.04)	8	0.47 (0.06)	6	0.55 (0.06)	7	5.3 (0.90)	5	6.6
**SPC**	0.73 (0.04)	9	0.99 (0.05)	1.5	0.49 (0.03)	9	0.65 (0.05)	14	0.65 (0.03)	5	0.47 (0.06)	8	0.55 (0.05)	4	5.3 (0.77)	9	7.4
**F-IAMB**	0.73 (0.04)	7	0.97 (0.08)	4	0.49 (0.05)	6	0.66 (0.07)	11	0.65 (0.04)	9	0.47 (0.06)	9	0.55 (0.06)	8	5.3 (0.89)	7	7.6
**Tabu**	0.54 (0.08)	10	0.09 (0.18)	10	0.08 (0.16)	10	0.68 (0.05)	3	0.30 (0.18)	10	−0.23 (0.22)	10	−0.23 (0.22)	10	9.3 (1.6)	10	9.1
**H2PC**	0.53 (0.06)	11	0.05 (0.11)	11.5	0.05 (0.11)	11	0.68 (0.04)	4	0.24 (0.12)	11	−0.26 (0.15)	11	−0.26 (0.15)	11	9.5 (1.1)	11	10.2
**MMHC**	0.52 (0.06)	12	0.05 (0.11)	13.5	0.05 (0.11)	12.5	0.68 (0.04)	5	0.23 (0.11)	12.5	−0.26 (0.15)	12	−0.26 (0.15)	12	9.5 (1.1)	12	11.4
**RSMAX2**	0.52 (0.06)	13	0.05 (0.11)	13.5	0.05 (0.11)	12.5	0.68 (0.04)	6	0.23 (0.11)	12.5	−0.26 (0.15)	13	−0.26 (0.15)	13	9.5 (1.1)	13	12.1
**HC**	0.52 (0.06)	14	0.05 (0.11)	11.5	0.05 (0.11)	14	0.68 (0.05)	7	0.23 (0.11)	14	−0.27 (0.15)	14	−0.27 (0.15)	14	9.6 (1.2)	14	12.8

Values in parentheses indicate standard deviations. F-IAMB: Fast-IAMB; GS: Grow-Shrink; H2PC: Hybrid HPC; HC: Hill-Climbing; HPC: Hybrid Parents and Children; IAMB: Incremental association Markov Blanket; IAMB-F: IAMB with False Discovery Rate Correction; I-IAMB: Interleaved IAMB; MMHC: Max–Min Hill-Climbing; MMPC: Max–Min Parents and Children; PCS: Peter–Clark Stable; RSMAX2: Restricted Maximization; SPC: Semi-Interleaved HITON-PC; Tabu: Tabu search. ACC: Accuracy; SEN: Sensitivity; PRE: Precision; SPE: Specificity; F1: F1 Score; CK: Cohen’s Kappa; MCC: Matthews Correlation Coefficient; SHD: Directed Structural Hamming Distance; R: Rank.

**Table A5:** Algorithm performance for DAG_5 with sample size *n* = 200.

	ACC	R_ACC_	SEN	R_SEN_	PRE	R_PRE_	SPE	R_SPE_	F1	R_F1_	CK	R_CK_	MCC	R_MCC_	SHD	R_SHD_	Overall Rank
**IAMB-F**	0.72 (0.09)	1	0.83 (0.23)	8	0.53 (0.15)	1	0.66 (0.15)	7	0.63 (0.14)	5	0.43 (0.20)	1	0.48 (0.20)	7	5.7 (1.9)	1	3.9
**GS**	0.69 (0.07)	3	0.92 (0.17)	7	0.50 (0.10)	3	0.59 (0.12)	8	0.63 (0.09)	4	0.40 (0.14)	4	0.48 (0.14)	4	6.3 (1.5)	3	4.5
**HPC**	0.68 (0.04)	7	0.96 (0.07)	3	0.49 (0.03)	7	0.56 (0.06)	12	0.64 (0.03)	1	0.41 (0.06)	2	0.49 (0.07)	1	6.4 (0.79)	7	5.0
**SPC**	0.68 (0.04)	8	0.97 (0.07)	2	0.48 (0.03)	8	0.55 (0.07)	13	0.64 (0.04)	2	0.40 (0.06)	3	0.49 (0.07)	2	6.5 (0.87)	8	5.8
**IAMB**	0.68 (0.07)	4.5	0.92 (0.17)	5.5	0.49 (0.10)	4.5	0.58 (0.12)	9.5	0.63 (0.09)	6.5	0.40 (0.14)	6.5	0.48 (0.14)	5.5	6.3 (1.4)	4.5	5.9
**I-IAMB**	0.68 (0.07)	4.5	0.92 (0.17)	5.5	0.49 (0.10)	4.5	0.58 (0.12)	9.5	0.63 (0.09)	6.5	0.40 (0.14)	6.5	0.48 (0.14)	5.5	6.3 (1.4)	4.5	5.9
**PCS**	0.70 (0.07)	2	0.71 (0.18)	9	0.50 (0.09)	2	0.70 (0.06)	6	0.58 (0.11)	9	0.36 (0.17)	9	0.38 (0.18)	9	6.0 (1.5)	2	6.0
**MMPC**	0.68 (0.04)	9	0.97 (0.07)	1	0.48 (0.03)	9	0.55 (0.07)	14	0.64 (0.04)	3	0.40 (0.06)	5	0.49 (0.07)	3	6.5 (0.88)	9	6.6
**F-IAMB**	0.68 (0.07)	6	0.92 (0.16)	4	0.49 (0.09)	6	0.58 (0.12)	11	0.63 (0.09)	8	0.40 (0.13)	8	0.48 (0.14)	8	6.4 (1.4)	6	7.1
**Tabu**	0.65 (0.13)	10	0.43 (0.20)	10	0.42 (0.21)	10	0.74 (0.10)	1	0.43 (0.20)	10	0.17 (0.30)	10	0.17 (0.30)	10	7.1 (2.6)	10	8.9
**RSMAX2**	0.62 (0.11)	11	0.37 (0.18)	13	0.37 (0.19)	11	0.73 (0.08)	3	0.38 (0.17)	13	0.10 (0.26)	11	0.10 (0.26)	11	7.6 (2.2)	11	10.5
**MMHC**	0.62 (0.11)	12	0.37 (0.18)	12	0.37 (0.19)	12	0.73 (0.08)	4	0.38 (0.17)	12	0.10 (0.26)	12	0.10 (0.26)	12	7.6 (2.2)	12	11.0
**H2PC**	0.62 (0.11)	13	0.36 (0.18)	14	0.36 (0.18)	13	0.73 (0.08)	2	0.38 (0.17)	14	0.09 (0.25)	13	0.09 (0.26)	13	7.6 (2.1)	13	11.9
**HC**	0.61 (0.12)	14	0.37 (0.19)	11	0.36 (0.19)	14	0.72 (0.09)	5	0.38 (0.18)	11	0.09 (0.27)	14	0.09 (0.27)	14	7.7 (2.4)	14	12.1

Values in parentheses indicate standard deviations. F-IAMB: Fast-IAMB; GS: Grow-Shrink; H2PC: Hybrid HPC; HC: Hill-Climbing; HPC: Hybrid Parents and Children; IAMB: Incremental association Markov Blanket; IAMB-F: IAMB with False Discovery Rate Correction; I-IAMB: Interleaved IAMB; MMHC: Max–Min Hill-Climbing; MMPC: Max–Min Parents and Children; PCS: Peter–Clark Stable; RSMAX2: Restricted Maximization; SPC: Semi-Interleaved HITON-PC; Tabu: Tabu search. ACC: Accuracy; SEN: Sensitivity; PRE: Precision; SPE: Specificity; F1: F1 Score; CK: Cohen’s Kappa; MCC: Matthews Correlation Coefficient; SHD: Directed Structural Hamming Distance; R: Rank.
