# Supplementary material for: Performance Evaluation of Bayesian Network Learning Algorithms in Structural Equation Modeling: A Simulation Study
Source: Entropy (Basel). 2026 Aug 25;28(9):955. doi: 10.3390/e28090955 (PMC13604978; doi:10.3390/e28090955)
Supplement: Supplementary file 1 [file entropy-28-00955-s001.zip › entropy-4499703-supplementary.pdf]

Table S1. Descriptive statistics of Goodness-of-Fit measures for DAG_1.

		Min	Max	Mean	Sd	Min	Max	Mean	Sd
**Goodness of fit Index**	**Suggested Threshold**	*n* =200				*n* =500			
**SRMR**	<=.08	.027	.050	.041	.004	.019	.043	.027	.004
**GFI**	>=.90	.929	.951	.942	.004	.964	.979	.975	.002
**AGFI**	>=.90	.900	.931	.918	.006	.950	.970	.965	.003
**CFI**	>=.90	.978	1.00	.994	.004	.989	1.00	.998	.002
**NFI**	>=.90	.933	.964	.953	.005	.971	.984	.981	.002
**NNFI**	>=.90	.973	1.00	.993	.005	.986	1.00	.997	.002
**IFI**	>=.90	.979	1.00	.994	.004	.989	1.00	.998	.002
**RMSEA**	<=.08	.001	.047	.024	.010	.001	.035	.015	.006
**Rel. ChiSq**	< 3	1.01	1.44	1.13	.093	1.01	1.59	1.13	.102
		n =1000				*n* =2500			
**SRMR**	<=.08	.012	.029	.019	.003	.008	.022	.012	.002
**GFI**	>=.90	.981	.989	.987	.001	.992	.996	.995	.000
**AGFI**	>=.90	.974	.985	.982	.002	.989	.994	.993	.001
**CFI**	>=.90	.994	1.00	.999	.001	.998	1.00	1.00	.000
**NFI**	>=.90	.986	.992	.990	.001	.994	.997	.996	.000
**NNFI**	>=.90	.993	1.00	.999	.001	.997	1.00	.999	.000
**IFI**	>=.90	.994	1.00	.999	.001	.998	1.00	1.00	.000
**RMSEA**	<=.08	.000	.026	.011	.005	.000	.017	.007	.003
**Rel. ChiSq**	< 3	1.01	1.68	1.13	.106	1.01	1.69	1.13	.110

Sd: Standard deviation; SRMR: Standardized Root Mean Squared Residual; GFI: Goodness-of-Fit Index; AGFI: Adjusted Goodness-of-Fit Index; CFI: Comparative Fit Index; NFI: Normed Fit Index; NNFI: Non-normed Fit Index; IFI: Incremental Fit Index; RMSEA: Root Mean Square Error of Approximation; Rel. ChiSq: Relative Chi-Squared

Table S2. Descriptive statistics of Goodness-of-Fit measures for DAG_2.

Goodness of fit Index	Suggested Threshold	Min	Max	Mean	Sd	Min	Max	Mean	Sd
		n =200				*n* =500			
**SRMR**	<=.08	.021	.046	.032	.004	.014	.033	.020	.003
**GFI**	>=.90	.941	.964	.956	.004	.970	.985	.981	.002
**AGFI**	>=.90	.907	.943	.929	.007	.952	.976	.971	.004
**CFI**	>=.90	.983	1.00	.995	.004	.989	1.00	.998	.002
**NFI**	>=.90	.951	.976	.966	.004	.977	.990	.986	.002
**NNFI**	>=.90	.977	1.00	.993	.005	.985	1.00	.997	.002
**IFI**	>=.90	.983	1.00	.995	.004	.989	1.00	.998	.002
**RMSEA**	<=.08	.001	.050	.027	.011	.001	.043	.017	.008
**Rel. ChiSq**	< 3	1.00	1.50	1.17	.122	1.01	1.93	1.17	.143
		n =1000				*n* =2500			
**SRMR**	<=.08	.010	.026	.014	.002	.007	.016	.009	.001
**GFI**	>=.90	.986	.992	.991	.001	.994	.997	.996	.000
**AGFI**	>=.90	.977	.988	.985	.002	.990	.995	.994	.001
**CFI**	>=.90	.995	1.00	.999	.001	.998	1.00	1.00	.000
**NFI**	>=.90	.989	.994	.993	.001	.995	.998	.997	.000
**NNFI**	>=.90	.994	1.00	.999	.001	.997	1.00	.999	.000
**IFI**	>=.90	.995	1.00	.999	.001	.998	1.00	1.00	.000
**RMSEA**	<=.08	.000	.028	.012	.005	.000	.019	.007	.003
**Rel. ChiSq**	< 3	1.01	1.79	1.16	.136	1.00	1.91	1.17	.136

Sd: Standard deviation; SRMR: Standardized Root Mean Squared Residual; GFI: Goodness-of-Fit Index; AGFI: Adjusted Goodness-of-Fit Index; CFI: Comparative Fit Index; NFI: Normed Fit Index; NNFI: Non-normed Fit Index; IFI: Incremental Fit Index; RMSEA: Root Mean Square Error of Approximation; Rel. ChiSq: Relative Chi-Squared

Table S3. Descriptive statistics of Goodness-of-Fit measures for DAG_3.

		Min	Max	Mean	Sd	Min	Max	Mean	Sd
**Goodness of fit Index**	**Suggested Threshold**	*n* =200				*n* =500			
**SRMR**	<=.08	.022	.050	.036	.006	.013	.042	.024	.004
**GFI**	>=.90	.959	.975	.968	.003	.977	.989	.986	.002
**AGFI**	>=.90	.927	.955	.943	.006	.958	.981	.976	.004
**CFI**	>=.90	.985	1.00	.995	.003	.988	1.00	.998	.002
**NFI**	>=.90	.956	.980	.971	.004	.979	.991	.988	.002
**NNFI**	>=.90	.978	1.00	.993	.005	.983	1.00	.996	.003
**IFI**	>=.90	.985	1.00	.995	.003	.988	1.00	.998	.002
**RMSEA**	<=.08	.001	.050	.029	.012	.001	.049	.020	.009
**Rel. ChiSq**	< 3	1.01	1.50	1.20	.136	1.01	2.22	1.24	.199
		n =1000				*n* =2500			
**SRMR**	<=.08	.010	.032	.017	.003	.006	.018	.011	.002
**GFI**	>=.90	.988	.995	.993	.001	.995	.998	.997	.000
**AGFI**	>=.90	.979	.990	.988	.002	.990	.996	.995	.001
**CFI**	>=.90	.994	1.00	.999	.001	.997	1.00	1.00	.000
**NFI**	>=.90	.989	.995	.994	.001	.995	.998	.998	.000
**NNFI**	>=.90	.991	1.00	.998	.001	.996	1.00	.999	.001
**IFI**	>=.90	.994	1.00	.999	.001	.997	1.00	1.00	.000
**RMSEA**	<=.08	.000	.034	.014	.006	.000	.024	.009	.004
**Rel. ChiSq**	< 3	1.01	2.19	1.24	.198	1.01	2.49	1.24	.205

Sd: Standard deviation; SRMR: Standardized Root Mean Squared Residual; GFI: Goodness-of-Fit Index; AGFI: Adjusted Goodness-of-Fit Index; CFI: Comparative Fit Index; NFI: Normed Fit Index; NNFI: Non-normed Fit Index; IFI: Incremental Fit Index; RMSEA: Root Mean Square Error of Approximation; Rel. ChiSq: Relative Chi-Squared

Table S4. Descriptive statistics of Goodness-of-Fit measures for DAG_4.

		Min	Max	Mean	Sd	Min	Max	Mean	Sd
**Goodness of fit Index**	**Suggested Threshold**	*n* =200				*n* =500			
**SRMR**	<=.08	.029	.050	.041	.005	.018	.046	.027	.004
**GFI**	>=.90	.929	.951	.942	.005	.965	.979	.975	.002
**AGFI**	>=.90	.900	.931	.918	.007	.951	.970	.965	.003
**CFI**	>=.90	.981	1.00	.994	.004	.990	1.00	.998	.002
**NFI**	>=.90	.937	.964	.954	.005	.973	.984	.981	.002
**NNFI**	>=.90	.976	1.00	.993	.005	.987	1.00	.997	.002
**IFI**	>=.90	.981	1.00	.994	.004	.990	1.00	.998	.002
**RMSEA**	<=.08	.001	.047	.024	.010	.001	.035	.015	.007
**Rel. ChiSq**	< 3	1.00	1.44	1.13	.097	1.00	1.61	1.14	.106
		n =1000				*n* =2500			
**SRMR**	<=.08	.012	.033	.019	.003	.008	.019	.012	.002
**GFI**	>=.90	.981	.989	.987	.001	.993	.996	.995	.000
**AGFI**	>=.90	.974	.985	.982	.002	.990	.994	.993	.001
**CFI**	>=.90	.994	1.00	.999	.001	.998	1.00	1.00	.000
**NFI**	>=.90	.986	.992	.990	.001	.995	.997	.996	.000
**NNFI**	>=.90	.993	1.00	.999	.001	.998	1.00	.999	.000
**IFI**	>=.90	.994	1.00	.999	.001	.998	1.00	1.00	.000
**RMSEA**	<=.08	.001	.026	.011	.005	.000	.014	.006	.003
**Rel. ChiSq**	< 3	1.00	1.69	1.13	.106	1.001	1.52	1.12	.098

Sd: Standard deviation; SRMR: Standardized Root Mean Squared Residual; GFI: Goodness-of-Fit Index; AGFI: Adjusted Goodness-of-Fit Index; CFI: Comparative Fit Index; NFI: Normed Fit Index; NNFI: Non-normed Fit Index; IFI: Incremental Fit Index; RMSEA: Root Mean Square Error of Approximation; Rel. ChiSq: Relative Chi-Squared

Table S5. Descriptive statistics of Goodness-of-Fit measures for DAG_5.

Goodness of fit Index	Suggested Threshold	Min	Max	Mean	Sd	Min	Max	Mean	Sd
		n =200				*n* =500			
**SRMR**	<=.08	.027	.050	.037	.004	.017	.037	.024	.003
**GFI**	>=.90	.930	.951	.942	.005	.965	.979	.976	.002
**AGFI**	>=.90	.900	.930	.917	.007	.950	.970	.965	.003
**CFI**	>=.90	.981	1.00	.994	.004	.989	1.00	.998	.002
**NFI**	>=.90	.938	.966	.956	.004	.973	.986	.982	.002
**NNFI**	>=.90	.977	1.00	.993	.005	.987	1.00	.997	.002
**IFI**	>=.90	.982	1.00	.994	.004	.989	1.00	.998	.002
**RMSEA**	<=.08	.002	.048	.024	.010	.001	.036	.015	.007
**Rel. ChiSq**	< 3	1.001	1.452	1.136	.096	1.00	1.635	1.131	.108
		n =1000				*n* =2500			
**SRMR**	<=.08	.012	.027	.017	.002	.008	.016	.011	.001
**GFI**	>=.90	.981	.989	.988	.001	.993	.996	.995	.000
**AGFI**	>=.90	.973	.985	.982	.002	.990	.994	.993	.001
**CFI**	>=.90	.994	1.00	.999	.001	.998	1.00	1.00	.000
**NFI**	>=.90	.986	.992	.991	.001	.995	.997	.996	.000
**NNFI**	>=.90	.992	1.00	.999	.001	.998	1.00	.999	.000
**IFI**	>=.90	.994	1.00	.999	.001	.998	1.00	1.00	.000
**RMSEA**	<=.08	.001	.028	.011	.005	.000	.015	.007	.003
**Rel. ChiSq**	< 3	1.001	1.782	1.137	.107	1.00	1.593	1.128	.103

Sd: Standard deviation; SRMR: Standardized Root Mean Squared Residual; GFI: Goodness-of-Fit Index; AGFI: Adjusted Goodness-of-Fit Index; CFI: Comparative Fit Index; NFI: Normed Fit Index; NNFI: Non-normed Fit Index; IFI: Incremental Fit Index; RMSEA: Root Mean Square Error of Approximation; Rel. ChiSq: Relative Chi-Squared

Table S6. Algorithm performance for DAG_1 with sample size *n* = 500.

	ACC	R_{ACC}	SEN	R_{SEN}	PRE	R_{PRE}	SPE	R_{SPE}	F1	R_{F1}	CK	R_{CK}	MCC	R_{MCC}	SHD	R_{SHD}	Overall Rank
PCS	.83 (.09)	1	.93 (.14)	4	.61 (.12)	1	.80 (.09)	1	.73 (.13)	1	.62 (.18)	1	.65 (.18)	1	3.4 (1.8)	1	1.4
HPC	.71 (.06)	2	1.00 (.01)	1	.47 (.04)	2	.62 (.07)	12	.64 (.04)	2	.45 (.07)	2	.54 (.06)	2	5.8 (1.1)	2	3.1
SPC	.71 (.06)	4	1.00 (.01)	2.5	.46 (.05)	3	.61 (.08)	13	.63 (.05)	3	.44 (.08)	3	.53 (.06)	3	5.9 (1.2)	4	4.4
MMPC	.70 (.06)	5	1.00 (.01)	2.5	.46 (.05)	4	.61 (.08)	14	.63 (.05)	4	.44 (.08)	4	.53 (.06)	4	5.9 (1.2)	5	5.3
IAMB-F	.71 (.06)	3	.82 (.26)	9	.44 (.08)	8	.67 (.08)	7	.57 (.13)	9	.37 (.16)	9	.43 (.19)	9	5.9 (1.2)	3	7.1
F-IAMB	.69 (.06)	12	.92 (.20)	5	.45 (.07)	5	.62 (.10)	11	.59 (.10)	5	.39 (.13)	5	.47 (.15)	5	6.1 (1.3)	12	7.5
IAMB	.69 (.06)	10.5	.91 (.20)	6.5	.44 (.07)	6.5	.62 (.10)	9.5	.59 (.10)	6.5	.39 (.13)	6.5	.47 (.15)	6.5	6.1 (1.3)	10.5	7.9
I-IAMB	.69 (.06)	10.5	.91 (.20)	6.5	.44 (.07)	6.5	.62 (.10)	9.5	.59 (.10)	6.5	.39 (.13)	6.5	.47 (.15)	6.5	6.1 (1.3)	10.5	7.9
H2PC	.70 (.16)	6	.42 (.30)	10	.40 (.29)	10	.79 (.11)	2	.44 (.29)	12	.21 (.40)	10	.21 (.40)	10	6.1 (3.1)	6	8.3
GS	.69 (.06)	9	.91 (.21)	8	.44 (.07)	9	.62 (.10)	8	.59 (.10)	8	.39 (.14)	8	.47 (.15)	8	6.1 (1.3)	9	8.4
RSMAX 2	.70 (.16)	7	.42 (.30)	11	.40 (.29)	11	.79 (.11)	3	.44 (.29)	11	.21 (.40)	11	.21 (.40)	11	6.1 (3.1)	7	9.0
MMHC	.69 (.16)	8	.42 (.30)	12	.40 (.29)	12	.79 (.11)	4	.44 (.29)	10	.20 (.40)	12	.20 (.40)	12	6.1 (3.1)	8	9.8
Tabu	.68 (.15)	13	.41 (.29)	13	.39 (.28)	13	.78 (.11)	5	.43 (.27)	14	.19 (.38)	13	.19 (.38)	13	6.3 (3.0)	13	12.1
HC	.68 (.16)	14	.41 (.31)	14	.38 (.29)	14	.77 (.12)	6	.44 (.28)	13	.18 (.41)	14	.18 (.41)	14	6.4 (3.2)	14	12.9

Values in parentheses indicate standard deviations.

F-IAMB: Fast-IAMB; GS: Grow-Shrink; H2PC: Hybrid HPC; HC: Hill-Climbing; HPC: Hybrid Parents and Children; IAMB: Incremental association Markov Blanket; IAMB-F: IAMB with False Discovery Rate Correction; I-IAMB: Interleaved IAMB; MMHC: Max-Min Hill-Climbing; MMPC: Max-Min Parents and Children; PCS: Peter-Clark Stable; RSMAX2: Restricted Maximization; SPC: Semi-Interleaved HITON-PC; Tabu: Tabu search

ACC: Accuracy; SEN: Sensitivity; PRE: Precision; SPE: Specificity; F1: F1 Score; CK: Cohen's Kappa; MCC: Matthews Correlation Coefficient; SHD: Directed Structural Hamming Distance; R: Rank

Table S7. Algorithm performance for DAG_1 with sample size n = 1000.

	ACC	R_{ACC}	SEN	R_{SEN}	PRE	R_{PRE}	SPE	R_{SPE}	F1	R_{F1}	CK	R_{CK}	MCC	R_{MCC}	SHD	R_{SHD}	Overall Rank
PCS	.81	1	.95	4	.59	1	.77	1	.73	1	.60	1	.64	1	3.7	1	1.4
	(.09)		(.11)		(.11)		(.10)		(.11)		(.17)		(.16)		(1.8)		
HPC	.68	3	1.0	2	.45	2	.58	12	.62	2	.41	2	.51	2	6.4	3	3.5
	(.07)		(.00)		(.05)		(.09)		(.05)		(.09)		(.07)		(1.3)		
SPC	.68	8	1.0	2	.44	3	.57	13	.61	3	.40	3	.50	3	6.5	8	5.4
	(.07)		(.00)		(.05)		(.09)		(.05)		(.09)		(.07)		(1.4)		
F-IAMB	.68	7	.88	5	.43	5	.61	11	.57	5	.36	5	.43	5	6.4	7	6.3
	(.09)		(.23)		(.10)		(.13)		(.13)		(.17)		(.18)		(1.8)		
MMPC	.67	9	1.0	2	.44	4	.56	14	.61	4	.40	4	.50	4	6.5	9	6.3
	(.07)		(.00)		(.05)		(.09)		(.05)		(.09)		(.07)		(1.4)		
IAMB	.68	5.5	.87	6.5	.43	6.5	.62	9.5	.57	6.5	.36	6.5	.43	6.5	6.4	5.5	6.6
	(.09)		(.24)		(.10)		(.13)		(.13)		(.17)		(.19)		(1.8)		
I-IAMB	.68	5.5	.87	6.5	.43	6.5	.62	9.5	.57	6.5	.36	6.5	.43	6.5	6.4	5.5	6.6
	(.09)		(.24)		(.10)		(.13)		(.13)		(.17)		(.19)		(1.8)		
IAMB-F	.69	2	.75	9	.43	9	.68	7	.54	9	.33	9	.38	9	6.1	2	7.0
	(.08)		(.28)		(.11)		(.11)		(.15)		(.20)		(.22)		(1.7)		
GS	.68	4	.86	8	.43	8	.62	8	.56	8	.36	8	.42	8	6.4	4	7.0
	(.09)		(.25)		(.10)		(.13)		(.13)		(.18)		(.19)		(1.8)		
H2PC	.66	10	.37	13	.34	10	.76	2	.38	12	.13	10	.13	10	6.8	10	9.6
	(.15)		(.29)		(.27)		(.11)		(.27)		(.38)		(.38)		(3.0)		
RSMAX2	.66	11	.37	11.5	.34	11	.76	3	.38	13	.13	11	.13	11	6.8	11	10.3
	(.15)		(.29)		(.27)		(.11)		(.27)		(.38)		(.38)		(3.0)		
MMHC	.66	12	.37	11.5	.34	12	.76	4	.38	14	.13	12	.13	12	6.8	12	11.2
	(.15)		(.29)		(.27)		(.11)		(.27)		(.38)		(.38)		(3.0)		
Tabu	.66	13	.37	10	.34	13	.75	5	.39	10	.12	13	.12	13	6.9	13	11.3
	(.15)		(.28)		(.26)		(.11)		(.26)		(.37)		(.37)		(3.0)		
HC	.65	14	.36	14	.33	14	.74	6	.39	11	.10	14	.10	14	7.0	14	12.6
	(.15)		(.29)		(.27)		(.11)		(.27)		(.39)		(.39)		(3.1)		

Values in parentheses indicate standard deviations.

F-IAMB: Fast-IAMB; GS: Grow-Shrink; H2PC: Hybrid HPC; HC: Hill-Climbing; HPC: Hybrid Parents and Children; IAMB: Incremental association Markov Blanket; IAMB-F: IAMB with False Discovery Rate Correction; I-IAMB: Interleaved IAMB; MMHC: Max-Min Hill-Climbing; MMPC: Max-Min Parents and Children; PCS: Peter-Clark Stable; RSMAX2: Restricted Maximization; SPC: Semi-Interleaved HITON-PC; Tabu: Tabu search

ACC: Accuracy; SEN: Sensitivity; PRE: Precision; SPE: Specificity; F1: F1 Score; CK: Cohen's Kappa; MCC: Matthews Correlation Coefficient; SHD: Directed Structural Hamming Distance; R: Rank

Table S8. Algorithm performance for DAG_1 with sample size n = 2500.

	ACC	R_{ACC}	SEN	R_{SEN}	PRE	R_{PRE}	SPE	R_{SPE}	F1	R_{F1}	CK	R_{CK}	MCC	R_{MCC}	SHD	R_{SHD}	Overall Rank
PCS	.76	1	.95	4	.53	1	.70	1	.68	1	.52	1	.57	1	4.8	1	1.4
	(.10)		(.10)		(.11)		(.13)		(.11)		(.17)		(.16)		(2.1)		
F-IAMB	.60	3	.88	5	.38	2	.51	8	.52	5	.27	5	.35	5	8.0	3	4.5
	(.12)		(.22)		(.11)		(.18)		(.12)		(.18)		(.19)		(2.4)		
SPC	.58	10	1.0	2	.38	5	.44	12	.55	2	.29	2	.41	2	8.4	10	5.6
	(.07)		(.00)		(.04)		(.09)		(.04)		(.08)		(.07)		(1.4)		
IAMB	.60	5.5	.87	6.5	.38	3.5	.50	10.5	.52	6.5	.26	6.5	.35	6.5	8.1	5.5	6.4
	(.12)		(.22)		(.10)		(.18)		(.12)		(.18)		(.19)		(2.4)		
I-IAMB	.60	5.5	.87	6.5	.38	3.5	.50	10.5	.52	6.5	.26	6.5	.35	6.5	8.1	5.5	6.4
	(.12)		(.22)		(.10)		(.18)		(.12)		(.18)		(.19)		(2.4)		
MMPC	.58	11	1.0	2	.38	7	.44	13	.55	3	.29	3	.41	3	8.4	11	6.6
	(.07)		(.00)		(.04)		(.09)		(.04)		(.08)		(.06)		(1.4)		
IAMB-F	.62	2	.76	9	.38	8	.57	7	.49	9	.24	9	.30	9	7.7	2	6.9
	(.11)		(.27)		(.11)		(.17)		(.14)		(.20)		(.21)		(2.2)		
GS	.60	4	.86	8	.38	6	.51	9	.52	8	.26	8	.34	8	8.1	4	6.9
	(.12)		(.23)		(.10)		(.18)		(.12)		(.18)		(.19)		(2.4)		
HPC	.57	14	1.0	2	.37	9	.42	14	.54	4	.27	4	.39	4	8.7	14	8.1
	(.08)		(.00)		(.05)		(.11)		(.05)		(.09)		(.08)		(1.7)		
Tabu	.58	9	.30	10	.24	10	.68	5	.30	10	-0.02	10	-0.02	10	8.4	9	9.1
	(.13)		(.24)		(.20)		(.10)		(.21)		(.31)		(.31)		(2.7)		
RSMAX2	.58	7	.29	11	.24	11	.68	2	.29	14	-0.03	11	-0.03	11	8.3	7	9.3
	(.13)		(.25)		(.20)		(.10)		(.21)		(.31)		(.31)		(2.6)		
MMHC	.58	8	.29	12	.24	12	.68	3	.29	13	-0.03	12	-0.03	12	8.3	8	10.0
	(.13)		(.25)		(.20)		(.10)		(.21)		(.31)		(.31)		(2.6)		
H2PC	.58	12	.29	13	.23	13	.68	4	.29	12	-0.03	13	-0.03	13	8.4	12	11.5
	(.13)		(.25)		(.20)		(.10)		(.22)		(.31)		(.32)		(2.7)		
HC	.57	13	.28	14	.23	14	.67	6	.29	11	-0.04	14	-0.04	14	8.6	13	12.4
	(.14)		(.25)		(.20)		(.11)		(.22)		(.32)		(.32)		(2.7)		

Values in parentheses indicate standard deviations.

F-IAMB: Fast-IAMB; GS: Grow-Shrink; H2PC: Hybrid HPC; HC: Hill-Climbing; HPC: Hybrid Parents and Children; IAMB: Incremental association Markov Blanket; IAMB-F: IAMB with False Discovery Rate Correction; I-IAMB: Interleaved IAMB; MMHC: Max-Min Hill-Climbing; MMPC: Max-Min Parents and Children; PCS: Peter-Clark Stable; RSMAX2: Restricted Maximization; SPC: Semi-Interleaved HITON-PC; Tabu: Tabu search

ACC: Accuracy; SEN: Sensitivity; PRE: Precision; SPE: Specificity; F1: F1 Score; CK: Cohen's Kappa; MCC: Matthews Correlation Coefficient; SHD: Directed Structural Hamming Distance; R: Rank

Table S9. Algorithm performance for DAG_2 with sample size n = 500.

	ACC	R_{ACC}	SEN	R_{SEN}	PRE	R_{PRE}	SPE	R_{SPE}	F1	R_{F1}	CK	R_{CK}	MCC	R_{MCC}	SHD	R_{SHD}	Overall Rank
HPC	.56	1	1.00	3	.49	1	.25	7	.66	1	.21	1	.38	7	5.3	1	2.8
	(.06)		(.02)		(.03)		(.10)		(.03)		(.09)		(.03)		(.69)		
SPC	.55	7.5	1.00	1.5	.48	6.5	.23	13.5	.65	2.5	.20	2.5	.38	1.5	5.4	7.5	5.4
	(.07)		(.02)		(.03)		(.12)		(.03)		(.10)		(.01)		(.81)		
IAMB	.55	4.5	1.00	5.5	.48	3.5	.23	9.5	.65	5.5	.20	5.5	.38	4.5	5.4	4.5	5.4
	(.07)		(.04)		(.04)		(.12)		(.03)		(.10)		(.02)		(.81)		
I-IAMB	.55	4.5	1.00	5.5	.48	3.5	.23	9.5	.65	5.5	.20	5.5	.38	4.5	5.4	4.5	5.4
	(.07)		(.04)		(.04)		(.12)		(.03)		(.10)		(.02)		(.81)		
F-IAMB	.55	4.5	1.00	5.5	.48	3.5	.23	9.5	.65	5.5	.20	5.5	.38	4.5	5.4	4.5	5.4
	(.07)		(.04)		(.04)		(.12)		(.03)		(.10)		(.02)		(.81)		
MMPC	.55	7.5	1.00	1.5	.48	6.5	.23	13.5	.65	2.5	.20	2.5	.38	1.5	5.4	7.5	5.4
	(.07)		(.02)		(.03)		(.12)		(.03)		(.10)		(.01)		(.81)		
GS	.55	4.5	1.00	5.5	.48	3.5	.23	9.5	.65	5.5	.20	5.5	.38	4.5	5.4	4.5	5.4
	(.07)		(.04)		(.04)		(.12)		(.03)		(.10)		(.02)		(.81)		
IAMB-F	.55	2	.96	8	.48	8	.26	6	.64	8	.19	8	.34	8	5.4	2	6.3
	(.07)		(.15)		(.05)		(.12)		(.08)		(.13)		(.15)		(.82)		
Tabu	.34	10	.22	10	.22	10	.43	1	.23	10	-0.35	10	-0.35	10	7.9	10	8.9
	(.09)		(.10)		(.10)		(.08)		(.09)		(.18)		(.18)		(1.1)		
PCS	.51	9	.90	9	.45	9	.23	12	.59	9	.11	9	.23	9	5.9	9	9.4
	(.10)		(.22)		(.08)		(.12)		(.12)		(.21)		(.28)		(1.2)		
H2PC	.34	11	.21	11	.21	11	.43	2	.22	14	-0.36	11	-0.36	11	8.0	11	10.3
	(.08)		(.09)		(.09)		(.08)		(.08)		(.16)		(.16)		(.94)		
MMHC	.33	12.5	.21	12.5	.21	12.5	.42	3.5	.22	12.5	-0.37	12.5	-0.37	12.5	8.0	12.5	11.4
	(.08)		(.09)		(.09)		(.08)		(.08)		(.16)		(.16)		(.98)		
RSMAX2	.33	12.5	.21	12.5	.21	12.5	.42	3.5	.22	12.5	-0.37	12.5	-0.37	12.5	8.0	12.5	11.4
	(.08)		(.09)		(.09)		(.08)		(.08)		(.16)		(.16)		(.98)		
HC	.33	14	.21	14	.20	14	.42	5	.22	11	-0.38	14	-0.38	14	8.1	14	12.5
	(.09)		(.10)		(.10)		(.09)		(.08)		(.18)		(.18)		(1.1)		

Values in parentheses indicate standard deviations.

F-IAMB: Fast-IAMB; GS: Grow-Shrink; H2PC: Hybrid HPC; HC: Hill-Climbing; HPC: Hybrid Parents and Children; IAMB: Incremental association Markov Blanket; IAMB-F: IAMB with False Discovery Rate Correction; I-IAMB: Interleaved IAMB; MMHC: Max-Min Hill-Climbing; MMPC: Max-Min Parents and Children; PCS: Peter-Clark Stable; RSMAX2: Restricted Maximization; SPC: Semi-Interleaved HITON-PC; Tabu: Tabu search

ACC: Accuracy; SEN: Sensitivity; PRE: Precision; SPE: Specificity; F1: F1 Score; CK: Cohen's Kappa; MCC: Matthews Correlation Coefficient; SHD: Directed Structural Hamming Distance; R: Rank

Table S10. Algorithm performance for DAG_2 with sample size n = 1000.

	ACC	R_{ACC}	SEN	R_{SEN}	PRE	R_{PRE}	SPE	R_{SPE}	F1	R_{F1}	CK	R_{CK}	MCC	R_{MCC}	SHD	R_{SHD}	Overall Rank
HPC	.54	1	1.0	2	.48	1	.22	7	.65	1	.19	1	.38	2	5.5	1	2.0
	(.07)		(.00)		(.04)		(.12)		(.03)		(.11)		(.00)		(.84)		
SPC	.53	2.5	1.0	2	.47	2.5	.20	11	.64	2.5	.17	2.5	.38	2	5.6	2.5	3.4
	(.08)		(.00)		(.04)		(.13)		(.04)		(.12)		(.00)		(.93)		
MMPC	.53	2.5	1.0	2	.47	2.5	.20	11	.64	2.5	.17	2.5	.38	2	5.6	2.5	3.4
	(.08)		(.00)		(.04)		(.13)		(.04)		(.12)		(.00)		(.93)		
IAMB	.53	6	1.00	5.5	.47	5.5	.20	11	.64	5.5	.17	5.5	.38	5.5	5.6	6	6.3
	(.08)		(.02)		(.04)		(.13)		(.04)		(.12)		(.03)		(.93)		
I-IAMB	.53	6	1.00	5.5	.47	5.5	.20	11	.64	5.5	.17	5.5	.38	5.5	5.6	6	6.3
	(.08)		(.02)		(.04)		(.13)		(.04)		(.12)		(.03)		(.93)		
F-IAMB	.53	6	1.00	5.5	.47	5.5	.20	11	.64	5.5	.17	5.5	.38	5.5	5.6	6	6.3
	(.08)		(.02)		(.04)		(.13)		(.04)		(.12)		(.03)		(.93)		
GS	.53	6	1.00	5.5	.47	5.5	.20	11	.64	5.5	.17	5.5	.38	5.5	5.6	6	6.3
	(.08)		(.02)		(.04)		(.13)		(.04)		(.12)		(.03)		(.93)		
IAMB-F	.53	6	.95	9	.47	9	.23	6	.62	9	.16	9	.32	9	5.6	6	7.9
	(.08)		(.17)		(.05)		(.13)		(.09)		(.14)		(.17)		(.93)		
PCS	.53	9	.99	8	.47	8	.20	11	.63	8	.16	8	.36	8	5.7	9	8.6
	(.08)		(.09)		(.05)		(.13)		(.06)		(.13)		(.12)		(.99)		
Tabu	.32	10	.20	10	.20	10	.41	1	.20	10	-0.38	10	-0.38	10	8.1	10	8.9
	(.05)		(.05)		(.05)		(.06)		(.04)		(.10)		(.10)		(.65)		
H2PC	.32	11	.20	11	.20	11	.41	2	.20	14	-0.39	11	-0.39	11	8.1	11	10.3
	(.05)		(.05)		(.05)		(.07)		(.04)		(.10)		(.10)		(.65)		
MMHC	.32	12.5	.20	12.5	.20	12.5	.41	3.5	.20	12.5	-0.39	12.5	-0.39	12.5	8.1	12.5	11.4
	(.05)		(.05)		(.05)		(.07)		(.04)		(.10)		(.10)		(.65)		
RSMAX2	.32	12.5	.20	12.5	.20	12.5	.41	3.5	.20	12.5	-0.39	12.5	-0.39	12.5	8.1	12.5	11.4
	(.05)		(.05)		(.05)		(.07)		(.04)		(.10)		(.10)		(.65)		
HC	.32	14	.20	14	.20	14	.41	5	.20	11	-0.39	14	-0.39	14	8.1	14	12.5
	(.06)		(.05)		(.05)		(.07)		(.04)		(.10)		(.11)		(.67)		

Values in parentheses indicate standard deviations.

F-IAMB: Fast-IAMB; GS: Grow-Shrink; H2PC: Hybrid HPC; HC: Hill-Climbing; HPC: Hybrid Parents and Children; IAMB: Incremental association Markov Blanket; IAMB-F: IAMB with False Discovery Rate Correction; I-IAMB: Interleaved IAMB; MMHC: Max-Min Hill-Climbing; MMPC: Max-Min Parents and Children; PCS: Peter-Clark Stable; RSMAX2: Restricted Maximization; SPC: Semi-Interleaved HITON-PC; Tabu: Tabu search

ACC: Accuracy; SEN: Sensitivity; PRE: Precision; SPE: Specificity; F1: F1 Score; CK: Cohen's Kappa; MCC: Matthews Correlation Coefficient; SHD: Directed Structural Hamming Distance; R: Rank

Table S11. Algorithm performance for DAG_2 with sample size n = 2500.

	ACC	R_{ACC}	SEN	R_{SEN}	PRE	R_{PRE}	SPE	R_{SPE}	F1	R_{F1}	CK	R_{CK}	MCC	R_{MCC}	SHD	R_{SHD}	Overall Rank
HPC	.49 (.08)	1	1.0 (.00)	4.5	.45 (.04)	1	.13 (.14)	7	.62 (.04)	1	.11 (.12)	1	.38 (.00)	4.5	6.1 (1.0)	1	2.6
PCS	.48 (.08)	5.5	1.0 (.00)	4.5	.45 (.04)	5	.11 (.14)	11	.62 (.04)	5	.09 (.12)	5	.38 (.00)	4.5	6.3 (.97)	5.5	5.8
SPC	.48 (.08)	5.5	1.0 (.00)	4.5	.45 (.04)	5	.11 (.14)	11	.62 (.04)	5	.09 (.12)	5	.38 (.00)	4.5	6.3 (.97)	5.5	5.8
IAMB	.48 (.08)	5.5	1.0 (.00)	4.5	.45 (.04)	5	.11 (.14)	11	.62 (.04)	5	.09 (.12)	5	.38 (.00)	4.5	6.3 (.97)	5.5	5.8
I-IAMB	.48 (.08)	5.5	1.0 (.00)	4.5	.45 (.04)	5	.11 (.14)	11	.62 (.04)	5	.09 (.12)	5	.38 (.00)	4.5	6.3 (.97)	5.5	5.8
F-IAMB	.48 (.08)	5.5	1.0 (.00)	4.5	.45 (.04)	5	.11 (.14)	11	.62 (.04)	5	.09 (.12)	5	.38 (.00)	4.5	6.3 (.97)	5.5	5.8
MMPC	.48 (.08)	5.5	1.0 (.00)	4.5	.45 (.04)	5	.11 (.14)	11	.62 (.04)	5	.09 (.12)	5	.38 (.00)	4.5	6.3 (.97)	5.5	5.8
GS	.48 (.08)	5.5	1.0 (.00)	4.5	.45 (.04)	5	.11 (.14)	11	.62 (.04)	5	.09 (.12)	5	.38 (.00)	4.5	6.3 (.97)	5.5	5.8
IAMB-F	.48 (.08)	5.5	.95 (.16)	9	.44 (.05)	9	.14 (.16)	6	.60 (.08)	9	.08 (.14)	9	.28 (.21)	9	6.3 (.97)	5.5	7.8
H2PC	.30 (.06)	12	.19 (.04)	12	.18 (.04)	12	.38 (.08)	3	.20 (.02)	12	-0.42 (.10)	12	-0.43 (.10)	12	8.4 (.68)	12	10.9
MMHC	.30 (.06)	12	.19 (.04)	12	.18 (.04)	12	.38 (.08)	3	.20 (.02)	12	-0.42 (.10)	12	-0.43 (.10)	12	8.4 (.68)	12	10.9
RSMAX2	.30 (.06)	12	.19 (.04)	12	.18 (.04)	12	.38 (.08)	3	.20 (.02)	12	-0.42 (.10)	12	-0.43 (.10)	12	8.4 (.68)	12	10.9
HC	.30 (.06)	12	.19 (.04)	12	.18 (.04)	12	.38 (.08)	3	.20 (.02)	12	-0.42 (.10)	12	-0.43 (.10)	12	8.4 (.68)	12	10.9
Tabu	.30 (.06)	12	.19 (.04)	12	.18 (.04)	12	.38 (.08)	3	.20 (.02)	12	-0.42 (.10)	12	-0.43 (.10)	12	8.4 (.68)	12	10.9

Values in parentheses indicate standard deviations.

F-IAMB: Fast-IAMB; GS: Grow-Shrink; H2PC: Hybrid HPC; HC: Hill-Climbing; HPC: Hybrid Parents and Children; IAMB: Incremental association Markov Blanket; IAMB-F: IAMB with False Discovery Rate Correction; I-IAMB: Interleaved IAMB; MMHC: Max-Min Hill-Climbing; MMPC: Max-Min Parents and Children; PCS: Peter-Clark Stable; RSMAX2: Restricted Maximization; SPC: Semi-Interleaved HITON-PC; Tabu: Tabu search

ACC: Accuracy; SEN: Sensitivity; PRE: Precision; SPE: Specificity; F1: F1 Score; CK: Cohen's Kappa; MCC: Matthews Correlation Coefficient; SHD: Directed Structural Hamming Distance; R: Rank

Table S12. Algorithm performance for DAG_3 with sample size n = 500.

	ACC	R_{ACC}	SEN	R_{SEN}	PRE	R_{PRE}	SPE	R_{SPE}	F1	R_{F1}	CK	R_{CK}	MCC	R_{MCC}	SHD	R_{SHD}	Overall Rank
IAMB-F	.61	6	.98	9	.47	6	.43	6	.63	5	.33	1	.49	9	2.3	6	6.0
	(.12)		(.09)		(.06)		(.19)		(.07)		(.15)		(.05)		(.73)		
H2PC	.65	3	.50	12	.48	3	.73	3	.49	12	.23	12	.23	12	2.1	3	7.5
	(.05)		(.00)		(.05)		(.07)		(.03)		(.07)		(.07)		(.29)		
MMHC	.65	3	.50	12	.48	3	.73	3	.49	12	.23	12	.23	12	2.1	3	7.5
	(.05)		(.00)		(.05)		(.07)		(.03)		(.07)		(.07)		(.29)		
RSMAX2	.65	3	.50	12	.48	3	.73	3	.49	12	.23	12	.23	12	2.1	3	7.5
	(.05)		(.00)		(.05)		(.07)		(.03)		(.07)		(.07)		(.29)		
HC	.65	3	.50	12	.48	3	.73	3	.49	12	.23	12	.23	12	2.1	3	7.5
	(.05)		(.00)		(.05)		(.07)		(.03)		(.07)		(.07)		(.29)		
Tabu	.65	3	.50	12	.48	3	.73	3	.49	12	.23	12	.23	12	2.1	3	7.5
	(.05)		(.00)		(.05)		(.07)		(.03)		(.07)		(.07)		(.29)		
PCS	.60	10.5	1.0	4.5	.47	10.5	.40	10.5	.63	5	.32	5.5	.50	4.5	2.4	10.5	7.7
	(.13)		(.00)		(.07)		(.20)		(.07)		(.16)		(.00)		(.79)		
HPC	.60	10.5	1.0	4.5	.47	10.5	.40	10.5	.63	5	.32	5.5	.50	4.5	2.4	10.5	7.7
	(.13)		(.00)		(.07)		(.20)		(.07)		(.16)		(.00)		(.79)		
SPC	.60	10.5	1.0	4.5	.47	10.5	.40	10.5	.63	5	.32	5.5	.50	4.5	2.4	10.5	7.7
	(.13)		(.00)		(.07)		(.20)		(.07)		(.16)		(.00)		(.79)		
IAMB	.60	10.5	1.0	4.5	.47	10.5	.40	10.5	.63	5	.32	5.5	.50	4.5	2.4	10.5	7.7
	(.13)		(.00)		(.07)		(.20)		(.07)		(.16)		(.00)		(.79)		
I-IAMB	.60	10.5	1.0	4.5	.47	10.5	.40	10.5	.63	5	.32	5.5	.50	4.5	2.4	10.5	7.7
	(.13)		(.00)		(.07)		(.20)		(.07)		(.16)		(.00)		(.79)		
F-IAMB	.60	10.5	1.0	4.5	.47	10.5	.40	10.5	.63	5	.32	5.5	.50	4.5	2.4	10.5	7.7
	(.13)		(.00)		(.07)		(.20)		(.07)		(.16)		(.00)		(.79)		
MMPC	.60	10.5	1.0	4.5	.47	10.5	.40	10.5	.63	5	.32	5.5	.50	4.5	2.4	10.5	7.7
	(.13)		(.00)		(.07)		(.20)		(.07)		(.16)		(.00)		(.79)		
GS	.60	10.5	1.0	4.5	.47	10.5	.40	10.5	.63	5	.32	5.5	.50	4.5	2.4	10.5	7.7
	(.13)		(.00)		(.07)		(.20)		(.07)		(.16)		(.00)		(.79)		

Values in parentheses indicate standard deviations.

F-IAMB: Fast-IAMB; GS: Grow-Shrink; H2PC: Hybrid HPC; HC: Hill-Climbing; HPC: Hybrid Parents and Children; IAMB: Incremental association Markov Blanket; IAMB-F: IAMB with False Discovery Rate Correction; I-IAMB: Interleaved IAMB; MMHC: Max-Min Hill-Climbing; MMPC: Max-Min Parents and Children; PCS: Peter-Clark Stable; RSMAX2: Restricted Maximization; SPC: Semi-Interleaved HITON-PC; Tabu: Tabu search

ACC: Accuracy; SEN: Sensitivity; PRE: Precision; SPE: Specificity; F1: F1 Score; CK: Cohen's Kappa; MCC: Matthews Correlation Coefficient; SHD: Directed Structural Hamming Distance; R: Rank

Table S13. Algorithm performance for DAG_3 with sample size n = 1000.

	ACC	R_{ACC}	SEN	R_{SEN}	PRE	R_{PRE}	SPE	R_{SPE}	F1	R_{F1}	CK	R_{CK}	MCC	R_{MCC}	SHD	R_{SHD}	Overall Rank
IAMB-F	.60	6	.98	9	.47	6	.41	6	.62	5	.31	1	.49	9	2.4	6	6.0
	(.14)		(.10)		(.07)		(.21)		(.07)		(.16)		(.06)		(.81)		
H2PC	.65	3	.50	12	.48	3	.72	3	.49	12	.22	12	.22	12	2.1	3	7.5
	(.05)		(.00)		(.05)		(.08)		(.03)		(.08)		(.08)		(.31)		
MMHC	.65	3	.50	12	.48	3	.72	3	.49	12	.22	12	.22	12	2.1	3	7.5
	(.05)		(.00)		(.05)		(.08)		(.03)		(.08)		(.08)		(.31)		
RSMAX2	.65	3	.50	12	.48	3	.72	3	.49	12	.22	12	.22	12	2.1	3	7.5
	(.05)		(.00)		(.05)		(.08)		(.03)		(.08)		(.08)		(.31)		
HC	.65	3	.50	12	.48	3	.72	3	.49	12	.22	12	.22	12	2.1	3	7.5
	(.05)		(.00)		(.05)		(.08)		(.03)		(.08)		(.08)		(.31)		
Tabu	.65	3	.50	12	.48	3	.72	3	.49	12	.22	12	.22	12	2.1	3	7.5
	(.05)		(.00)		(.05)		(.08)		(.03)		(.08)		(.08)		(.31)		
PCS	.58	10.5	1.0	4.5	.46	10.5	.38	10.5	.62	5	.30	5.5	.50	4.5	2.5	10.5	7.7
	(.14)		(.00)		(.07)		(.22)		(.07)		(.17)		(.00)		(.87)		
HPC	.58	10.5	1.0	4.5	.46	10.5	.38	10.5	.62	5	.30	5.5	.50	4.5	2.5	10.5	7.7
	(.14)		(.00)		(.07)		(.22)		(.07)		(.17)		(.00)		(.87)		
SPC	.58	10.5	1.0	4.5	.46	10.5	.38	10.5	.62	5	.30	5.5	.50	4.5	2.5	10.5	7.7
	(.14)		(.00)		(.07)		(.22)		(.07)		(.17)		(.00)		(.87)		
IAMB	.58	10.5	1.0	4.5	.46	10.5	.38	10.5	.62	5	.30	5.5	.50	4.5	2.5	10.5	7.7
	(.14)		(.00)		(.07)		(.22)		(.07)		(.17)		(.00)		(.87)		
I-IAMB	.58	10.5	1.0	4.5	.46	10.5	.38	10.5	.62	5	.30	5.5	.50	4.5	2.5	10.5	7.7
	(.14)		(.00)		(.07)		(.22)		(.07)		(.17)		(.00)		(.87)		
F-IAMB	.58	10.5	1.0	4.5	.46	10.5	.38	10.5	.62	5	.30	5.5	.50	4.5	2.5	10.5	7.7
	(.14)		(.00)		(.07)		(.22)		(.07)		(.17)		(.00)		(.87)		
MMPC	.58	10.5	1.0	4.5	.46	10.5	.38	10.5	.62	5	.30	5.5	.50	4.5	2.5	10.5	7.7
	(.14)		(.00)		(.07)		(.22)		(.07)		(.17)		(.00)		(.87)		
GS	.58	10.5	1.0	4.5	.46	10.5	.38	10.5	.62	5	.30	5.5	.50	4.5	2.5	10.5	7.7
	(.14)		(.00)		(.07)		(.22)		(.07)		(.17)		(.00)		(.87)		

Values in parentheses indicate standard deviations.

F-IAMB: Fast-IAMB; GS: Grow-Shrink; H2PC: Hybrid HPC; HC: Hill-Climbing; HPC: Hybrid Parents and Children; IAMB: Incremental association Markov Blanket; IAMB-F: IAMB with False Discovery Rate Correction; I-IAMB: Interleaved IAMB; MMHC: Max-Min Hill-Climbing; MMPC: Max-Min Parents and Children; PCS: Peter-Clark Stable; RSMAX2: Restricted Maximization; SPC: Semi-Interleaved HITON-PC; Tabu: Tabu search

ACC: Accuracy; SEN: Sensitivity; PRE: Precision; SPE: Specificity; F1: F1 Score; CK: Cohen's Kappa; MCC: Matthews Correlation Coefficient; SHD: Directed Structural Hamming Distance; R: Rank

Table S14. Algorithm performance for DAG_3 with sample size n = 2500.

	ACC	R_{ACC}	SEN	R_{SEN}	PRE	R_{PRE}	SPE	R_{SPE}	F1	R_{F1}	CK	R_{CK}	MCC	R_{MCC}	SHD	R_{SHD}	Overall Rank
IAMB-F	.51 (.17)	6	.97 (.12)	9	.42 (.08)	6	.28 (.27)	6	.58 (.08)	5	.20 (.19)	1	.47 (.08)	9	3.0 (1.0)	6	6.0
H2PC	.63 (.07)	3	.50 (.00)	12	.46 (.07)	3	.69 (.11)	3	.48 (.04)	12	.19 (.11)	4	.19 (.11)	12	2.2 (.43)	3	6.5
MMHC	.63 (.07)	3	.50 (.00)	12	.46 (.07)	3	.69 (.11)	3	.48 (.04)	12	.19 (.11)	4	.19 (.11)	12	2.2 (.43)	3	6.5
RSMAX2	.63 (.07)	3	.50 (.00)	12	.46 (.07)	3	.69 (.11)	3	.48 (.04)	12	.19 (.11)	4	.19 (.11)	12	2.2 (.43)	3	6.5
HC	.63 (.07)	3	.50 (.00)	12	.46 (.07)	3	.69 (.11)	3	.48 (.04)	12	.19 (.11)	4	.19 (.11)	12	2.2 (.43)	3	6.5
Tabu	.63 (.07)	3	.50 (.00)	12	.46 (.07)	3	.69 (.11)	3	.48 (.04)	12	.19 (.11)	4	.19 (.11)	12	2.2 (.43)	3	6.5
PCS	.49 (.17)	10.5	1.0 (.00)	4.5	.41 (.08)	10.5	.23 (.25)	10.5	.58 (.08)	5	.18 (.20)	10.5	.50 (.00)	4.5	3.1 (1.0)	10.5	8.3
HPC	.49 (.17)	10.5	1.0 (.00)	4.5	.41 (.08)	10.5	.23 (.25)	10.5	.58 (.08)	5	.18 (.20)	10.5	.50 (.00)	4.5	3.1 (1.0)	10.5	8.3
SPC	.49 (.17)	10.5	1.0 (.00)	4.5	.41 (.08)	10.5	.23 (.25)	10.5	.58 (.08)	5	.18 (.20)	10.5	.50 (.00)	4.5	3.1 (1.0)	10.5	8.3
IAMB	.49 (.17)	10.5	1.0 (.00)	4.5	.41 (.08)	10.5	.23 (.25)	10.5	.58 (.08)	5	.18 (.20)	10.5	.50 (.00)	4.5	3.1 (1.0)	10.5	8.3
I-IAMB	.49 (.17)	10.5	1.0 (.00)	4.5	.41 (.08)	10.5	.23 (.25)	10.5	.58 (.08)	5	.18 (.20)	10.5	.50 (.00)	4.5	3.1 (1.0)	10.5	8.3
F-IAMB	.49 (.17)	10.5	1.0 (.00)	4.5	.41 (.08)	10.5	.23 (.25)	10.5	.58 (.08)	5	.18 (.20)	10.5	.50 (.00)	4.5	3.1 (1.0)	10.5	8.3
MMPC	.49 (.17)	10.5	1.0 (.00)	4.5	.41 (.08)	10.5	.23 (.25)	10.5	.58 (.08)	5	.18 (.20)	10.5	.50 (.00)	4.5	3.1 (1.0)	10.5	8.3
GS	.49 (.17)	10.5	1.0 (.00)	4.5	.41 (.08)	10.5	.23 (.25)	10.5	.58 (.08)	5	.18 (.20)	10.5	.50 (.00)	4.5	3.1 (1.0)	10.5	8.3

Values in parentheses indicate standard deviations.

F-IAMB: Fast-IAMB; GS: Grow-Shrink; H2PC: Hybrid HPC; HC: Hill-Climbing; HPC: Hybrid Parents and Children; IAMB: Incremental association Markov Blanket; IAMB-F: IAMB with False Discovery Rate Correction; I-IAMB: Interleaved IAMB; MMHC: Max-Min Hill-Climbing; MMPC: Max-Min Parents and Children; PCS: Peter-Clark Stable; RSMAX2: Restricted Maximization; SPC: Semi-Interleaved HITON-PC; Tabu: Tabu search

ACC: Accuracy; SEN: Sensitivity; PRE: Precision; SPE: Specificity; F1: F1 Score; CK: Cohen's Kappa; MCC: Matthews Correlation Coefficient; SHD: Directed Structural Hamming Distance; R: Rank

Table S15. Algorithm performance for DAG_4 with sample size n = 500.

	ACC	R_{ACC}	SEN	R_{SEN}	PRE	R_{PRE}	SPE	R_{SPE}	F1	R_{F1}	CK	R_{CK}	MCC	R_{MCC}	SHD	R_{SHD}	Overall Rank
IAMB-F	.74 (.04)	2	.93 (.14)	8	.49 (.05)	2	.68 (.08)	2	.64 (.05)	4	.47 (.07)	1	.53 (.08)	4	5.2 (.89)	2	3.1
PCS	.74 (.07)	1	.90 (.16)	9	.50 (.09)	1	.69 (.08)	1	.64 (.10)	5	.46 (.14)	2	.52 (.15)	9	5.2 (1.4)	1	3.6
HPC	.72 (.05)	5	1.0 (.00)	2	.47 (.04)	7	.62 (.07)	12	.64 (.04)	1	.46 (.07)	3	.54 (.05)	1	5.7 (1.0)	5	4.5
SPC	.72 (.05)	8	1.0 (.00)	2	.47 (.04)	8	.62 (.07)	13	.64 (.04)	2	.45 (.07)	4	.54 (.05)	2	5.7 (1.0)	8	5.9
F-IAMB	.72 (.06)	4	.97 (.10)	6	.48 (.05)	4	.63 (.09)	9	.63 (.05)	6	.45 (.08)	7	.53 (.07)	7	5.6 (1.1)	4	5.9
GS	.72 (.05)	3	.96 (.11)	7	.48 (.05)	3	.64 (.09)	8	.63 (.05)	9	.45 (.08)	6	.53 (.07)	8	5.6 (1.1)	3	5.9
MMPC	.71 (.05)	9	1.0 (.00)	2	.47 (.04)	9	.62 (.07)	14	.64 (.04)	3	.45 (.07)	5	.54 (.05)	3	5.7 (1.1)	9	6.8
IAMB	.72 (.06)	6.5	.97 (.09)	4.5	.47 (.05)	5.5	.63 (.09)	10.5	.63 (.05)	7.5	.45 (.08)	8.5	.53 (.07)	5.5	5.7 (1.1)	6.5	6.9
I-IAMB	.72 (.06)	6.5	.97 (.09)	4.5	.47 (.05)	5.5	.63 (.09)	10.5	.63 (.05)	7.5	.45 (.08)	8.5	.53 (.07)	5.5	5.7 (1.1)	6.5	6.9
Tabu	.51 (.07)	10	.06 (.15)	10	.06 (.12)	10	.66 (.05)	3	.28 (.15)	10	-0.27 (.18)	10	-0.27 (.18)	10	9.7 (1.4)	10	9.1
RSMAX2	.50 (.04)	11	.03 (.07)	12	.03 (.07)	11	.66 (.04)	4.5	.20 (.05)	12	-0.31 (.10)	11	-0.31 (.10)	11	10 (.83)	11	10.4
H2PC	.50 (.04)	12	.03 (.07)	14	.03 (.07)	13	.66 (.04)	4.5	.20 (.05)	11	-0.31 (.10)	13	-0.31 (.10)	13	10 (.83)	12	11.6
MMHC	.50 (.04)	13	.03 (.07)	12	.03 (.07)	12	.66 (.04)	6	.20 (.05)	13	-0.31 (.10)	12	-0.31 (.10)	12	10 (.83)	13	11.6
HC	.50 (.04)	14	.03 (.07)	12	.03 (.07)	14	.66 (.04)	7	.20 (.05)	14	-0.31 (.10)	14	-0.31 (.10)	14	10 (.85)	14	12.9

Values in parentheses indicate standard deviations.

F-IAMB: Fast-IAMB; GS: Grow-Shrink; H2PC: Hybrid HPC; HC: Hill-Climbing; HPC: Hybrid Parents and Children; IAMB: Incremental association Markov Blanket; IAMB-F: IAMB with False Discovery Rate Correction; I-IAMB: Interleaved IAMB; MMHC: Max-Min Hill-Climbing; MMPC: Max-Min Parents and Children; PCS: Peter-Clark Stable; RSMAX2: Restricted Maximization; SPC: Semi-Interleaved HITON-PC; Tabu: Tabu search

ACC: Accuracy; SEN: Sensitivity; PRE: Precision; SPE: Specificity; F1: F1 Score; CK: Cohen's Kappa; MCC: Matthews Correlation Coefficient; SHD: Directed Structural Hamming Distance; R: Rank

Table S16. Algorithm performance for DAG_4 with sample size n = 1000.

	ACC	R_{ACC}	SEN	R_{SEN}	PRE	R_{PRE}	SPE	R_{SPE}	F1	R_{F1}	CK	R_{CK}	MCC	R_{MCC}	SHD	R_{SHD}	Overall Rank
IAMB-F	.73	1	.89	9	.49	1	.68	1	.62	4	.44	1	.50	4	5.4	1	2.8
	(.06)		(.16)		(.06)		(.10)		(.06)		(.09)		(.10)		(1.1)		
PCS	.72	2	.92	8	.47	2	.66	2	.62	5	.43	2	.50	5	5.6	2	3.5
	(.07)		(.18)		(.08)		(.07)		(.10)		(.15)		(.17)		(1.4)		
HPC	.70	7	1.0	2	.46	7	.60	12	.63	1	.43	3	.52	1	6.1	7	5.0
	(.06)		(.00)		(.05)		(.08)		(.05)		(.08)		(.06)		(1.2)		
SPC	.70	8	1.0	2	.46	8	.59	13	.63	2	.43	4	.52	2	6.1	8	5.9
	(.06)		(.00)		(.05)		(.08)		(.05)		(.08)		(.06)		(1.3)		
IAMB	.70	4.5	.95	4.5	.46	4.5	.61	10.5	.61	6.5	.42	6.5	.50	6.5	6.0	4.5	6.0
	(.07)		(.12)		(.06)		(.11)		(.06)		(.09)		(.09)		(1.3)		
I-IAMB	.70	4.5	.95	4.5	.46	4.5	.61	10.5	.61	6.5	.42	6.5	.50	6.5	6.0	4.5	6.0
	(.07)		(.12)		(.06)		(.11)		(.06)		(.09)		(.09)		(1.3)		
GS	.70	3	.94	7	.46	3	.62	8	.61	8	.42	8	.50	9	6.0	3	6.1
	(.07)		(.14)		(.06)		(.11)		(.06)		(.09)		(.09)		(1.3)		
MMPC	.69	9	1.0	2	.46	9	.59	14	.62	3	.43	5	.52	3	6.1	9	6.8
	(.06)		(.00)		(.05)		(.08)		(.05)		(.08)		(.06)		(1.3)		
F-IAMB	.70	6	.95	6	.46	6	.62	9	.61	9	.42	9	.50	8	6.0	6	7.4
	(.07)		(.13)		(.06)		(.11)		(.06)		(.09)		(.09)		(1.3)		
Tabu	.49	10	.03	10	.03	10	.65	3	.33	10	-0.31	10	-0.31	10	10.1	10	9.1
	(.06)		(.12)		(.10)		(.05)		(.16)		(.14)		(.15)		(1.2)		
H2PC	.49	11	.01	12.5	.01	11	.64	4	.19	11	-0.34	11	-0.34	11	10.3	11	10.3
	(.03)		(.04)		(.04)		(.04)		(.01)		(.05)		(.06)		(.65)		
RSMAX2	.48	12	.01	12.5	.01	12	.64	5	.19	12	-0.34	12	-0.34	12	10.3	12	11.2
	(.03)		(.04)		(.04)		(.04)		(.01)		(.05)		(.05)		(.65)		
MMHC	.48	13	.01	12.5	.01	13	.64	6	.19	13	-0.34	13	-0.34	13	10.3	13	12.1
	(.03)		(.04)		(.04)		(.04)		(.01)		(.05)		(.05)		(.65)		
HC	.48	14	.01	12.5	.01	14	.64	7	.19	14	-0.34	14	-0.34	14	10.4	14	12.9
	(.03)		(.04)		(.04)		(.04)		(.01)		(.05)		(.06)		(.68)		

Values in parentheses indicate standard deviations.

F-IAMB: Fast-IAMB; GS: Grow-Shrink; H2PC: Hybrid HPC; HC: Hill-Climbing; HPC: Hybrid Parents and Children; IAMB: Incremental association Markov Blanket; IAMB-F: IAMB with False Discovery Rate Correction; I-IAMB: Interleaved IAMB; MMHC: Max-Min Hill-Climbing; MMPC: Max-Min Parents and Children; PCS: Peter-Clark Stable; RSMAX2: Restricted Maximization; SPC: Semi-Interleaved HITON-PC; Tabu: Tabu search

ACC: Accuracy; SEN: Sensitivity; PRE: Precision; SPE: Specificity; F1: F1 Score; CK: Cohen's Kappa; MCC: Matthews Correlation Coefficient; SHD: Directed Structural Hamming Distance; R: Rank

Table S17. Algorithm performance for DAG_4 with sample size n = 2500.

	ACC	R_{ACC}	SEN	R_{SEN}	PRE	R_{PRE}	SPE	R_{SPE}	F1	R_{F1}	CK	R_{CK}	MCC	R_{MCC}	SHD	R_{SHD}	Overall Rank
IAMB-F	.70	1	.83	9	.46	1	.65	1	.58	4	.38	1	.44	6	6.1	1	3.0
	(.08)		(.19)		(.08)		(.14)		(.08)		(.11)		(.11)		(1.6)		
HPC	.64	7	1.0	2	.42	7	.52	12	.59	1	.36	2	.46	1	7.2	7	4.9
	(.07)		(.00)		(.05)		(.10)		(.05)		(.09)		(.07)		(1.4)		
IAMB	.65	5.5	.92	4.5	.43	3.5	.56	10.5	.57	5.5	.35	5.5	.44	4.5	6.9	5.5	5.6
	(.09)		(.15)		(.07)		(.14)		(.06)		(.10)		(.09)		(1.7)		
I-IAMB	.65	5.5	.92	4.5	.43	3.5	.56	10.5	.57	5.5	.35	5.5	.44	4.5	6.9	5.5	5.6
	(.09)		(.15)		(.07)		(.14)		(.06)		(.10)		(.09)		(1.7)		
SPC	.64	8	1.0	2	.42	8	.52	13	.58	2	.36	3	.46	2	7.2	8	5.8
	(.07)		(.00)		(.05)		(.09)		(.05)		(.09)		(.07)		(1.4)		
GS	.66	3	.91	7	.43	2	.57	8	.57	8	.35	7	.43	8	6.9	3	5.8
	(.09)		(.16)		(.07)		(.15)		(.06)		(.10)		(.10)		(1.7)		
F-IAMB	.65	4	.91	6	.43	5	.57	9	.57	7	.35	8	.43	7	6.9	4	6.3
	(.08)		(.16)		(.07)		(.14)		(.06)		(.10)		(.09)		(1.7)		
PCS	.67	2	.87	8	.42	6	.60	7	.56	9	.35	9	.41	9	6.6	2	6.5
	(.09)		(.22)		(.10)		(.10)		(.13)		(.19)		(.21)		(1.8)		
MMPC	.64	9	1.0	2	.41	9	.52	14	.58	3	.35	4	.46	3	7.3	9	6.6
	(.07)		(.00)		(.05)		(.09)		(.05)		(.09)		(.07)		(1.4)		
Tabu	.47	10	.03	10	.02	10	.61	3	.34	10	-0.34	10	-0.34	10	10.7	10	9.1
	(.05)		(.10)		(.08)		(.05)		(.13)		(.12)		(.12)		(1.1)		
H2PC	.46	11	.00	13.5	.00	13	.61	2	.17	11	-0.36	11	-0.37	11	10.8	11	10.4
	(.03)		(.03)		(.02)		(.04)		(.01)		(.04)		(.04)		(.64)		
RSMAX2	.46	12	.00	11.5	.00	11.5	.61	4	.17	12.5	-0.36	12	-0.37	12	10.8	12	10.9
	(.03)		(.03)		(.02)		(.04)		(.01)		(.04)		(.04)		(.61)		
MMHC	.46	13	.00	11.5	.00	11.5	.61	5	.17	12.5	-0.36	13	-0.37	13	10.8	13	11.6
	(.03)		(.03)		(.02)		(.04)		(.01)		(.04)		(.04)		(.62)		
HC	.46	14	.00	13.5	.00	14	.61	6	.17	14	-0.37	14	-0.37	14	10.9	14	12.9
	(.03)		(.03)		(.02)		(.04)		(.00)		(.04)		(.04)		(.66)		

Values in parentheses indicate standard deviations.

F-IAMB: Fast-IAMB; GS: Grow-Shrink; H2PC: Hybrid HPC; HC: Hill-Climbing; HPC: Hybrid Parents and Children; IAMB: Incremental association Markov Blanket; IAMB-F: IAMB with False Discovery Rate Correction; I-IAMB: Interleaved IAMB; MMHC: Max-Min Hill-Climbing; MMPC: Max-Min Parents and Children; PCS: Peter-Clark Stable; RSMAX2: Restricted Maximization; SPC: Semi-Interleaved HITON-PC; Tabu: Tabu search

ACC: Accuracy; SEN: Sensitivity; PRE: Precision; SPE: Specificity; F1: F1 Score; CK: Cohen's Kappa; MCC: Matthews Correlation Coefficient; SHD: Directed Structural Hamming Distance; R: Rank

Table S18. Algorithm performance for DAG_5 with sample size n = 500.

	ACC	R_{ACC}	SEN	R_{SEN}	PRE	R_{PRE}	SPE	R_{SPE}	F1	R_{F1}	CK	R_{CK}	MCC	R_{MCC}	SHD	R_{SHD}	Overall Rank
PCS	.72	1	.83	8	.53	1	.68	6	.64	2	.43	1	.47	4	5.5	1	3.0
	(.09)		(.18)		(.09)		(.08)		(.12)		(.18)		(.20)		(1.8)		
HPC	.66	3	1.00	3	.47	2	.52	12	.64	1	.39	2	.49	1	6.8	3	3.4
	(.05)		(.01)		(.04)		(.08)		(.03)		(.07)		(.06)		(1.1)		
SPC	.65	8	1.0	1.5	.47	3	.51	13	.64	3	.38	3	.49	2	6.9	8	5.2
	(.06)		(.00)		(.04)		(.08)		(.04)		(.07)		(.06)		(1.1)		
IAMB	.66	4.5	.93	5.5	.47	5.5	.54	9.5	.62	5.5	.37	5.5	.45	5.5	6.8	4.5	5.8
	(.08)		(.20)		(.08)		(.12)		(.10)		(.15)		(.16)		(1.5)		
I-IAMB	.66	4.5	.93	5.5	.47	5.5	.54	9.5	.62	5.5	.37	5.5	.45	5.5	6.8	4.5	5.8
	(.08)		(.20)		(.08)		(.12)		(.10)		(.15)		(.16)		(1.5)		
MMPC	.65	9	1.0	1.5	.47	4	.51	14	.64	4	.38	4	.49	3	6.9	9	6.1
	(.06)		(.00)		(.04)		(.08)		(.04)		(.07)		(.06)		(1.1)		
IAMB-F	.67	2	.83	9	.46	9	.60	7	.58	9	.34	9	.41	9	6.7	2	7.0
	(.08)		(.28)		(.10)		(.12)		(.14)		(.19)		(.21)		(1.5)		
F-IAMB	.66	7	.93	4	.47	8	.54	11	.62	7	.37	7	.45	7	6.8	7	7.3
	(.08)		(.19)		(.07)		(.12)		(.10)		(.14)		(.15)		(1.5)		
GS	.66	6	.92	7	.47	7	.55	8	.61	8	.36	8	.45	8	6.8	6	7.3
	(.08)		(.20)		(.08)		(.12)		(.11)		(.15)		(.16)		(1.5)		
Tabu	.63	10	.42	10	.39	10	.72	1	.41	10	.14	10	.14	10	7.5	10	8.9
	(.11)		(.18)		(.17)		(.09)		(.17)		(.26)		(.26)		(2.3)		
H2PC	.60	11	.36	11	.35	11	.70	2	.36	11	.07	11	.07	11	8.0	11	9.9
	(.09)		(.14)		(.13)		(.07)		(.13)		(.19)		(.19)		(1.7)		
RSMAX2	.60	12	.36	12.5	.35	12	.70	3	.36	12	.06	12	.06	12	8.0	12	10.9
	(.09)		(.14)		(.13)		(.07)		(.13)		(.20)		(.20)		(1.7)		
MMHC	.60	13	.36	12.5	.34	13	.70	4	.36	13	.06	13	.06	13	8.0	13	11.8
	(.09)		(.14)		(.13)		(.07)		(.13)		(.20)		(.20)		(1.7)		
HC	.58	14	.36	14	.33	14	.68	5	.35	14	.04	14	.04	14	8.3	14	12.9
	(.10)		(.15)		(.14)		(.08)		(.14)		(.21)		(.22)		(1.9)		

Values in parentheses indicate standard deviations.

F-IAMB: Fast-IAMB; GS: Grow-Shrink; H2PC: Hybrid HPC; HC: Hill-Climbing; HPC: Hybrid Parents and Children; IAMB: Incremental association Markov Blanket; IAMB-F: IAMB with False Discovery Rate Correction; I-IAMB: Interleaved IAMB; MMHC: Max-Min Hill-Climbing; MMPC: Max-Min Parents and Children; PCS: Peter-Clark Stable; RSMAX2: Restricted Maximization; SPC: Semi-Interleaved HITON-PC; Tabu: Tabu search

ACC: Accuracy; SEN: Sensitivity; PRE: Precision; SPE: Specificity; F1: F1 Score; CK: Cohen's Kappa; MCC: Matthews Correlation Coefficient; SHD: Directed Structural Hamming Distance; R: Rank

Table S19. Algorithm performance for DAG_5 with sample size n = 1000.

	ACC	R_{ACC}	SEN	R_{SEN}	PRE	R_{PRE}	SPE	R_{SPE}	F1	R_{F1}	CK	R_{CK}	MCC	R_{MCC}	SHD	R_{SHD}	Overall Rank
PCS	.75	1	.91	8	.55	1	.68	2	.69	1	.50	1	.54	1	5.1	1	2.0
	(.09)		(.14)		(.09)		(.10)		(.11)		(.17)		(.18)		(1.9)		
IAMB	.68	3.5	.95	5.5	.49	2.5	.57	8.5	.64	2.5	.41	2.5	.49	2.5	6.4	3.5	3.9
	(.11)		(.16)		(.10)		(.16)		(.11)		(.18)		(.17)		(2.2)		
I-IAMB	.68	3.5	.95	5.5	.49	2.5	.57	8.5	.64	2.5	.41	2.5	.49	2.5	6.4	3.5	3.9
	(.11)		(.16)		(.10)		(.16)		(.11)		(.18)		(.17)		(2.2)		
F-IAMB	.68	5	.95	4	.49	5	.56	11	.64	4	.41	4	.49	4	6.4	5	5.3
	(.11)		(.16)		(.10)		(.16)		(.11)		(.18)		(.17)		(2.2)		
IAMB-F	.69	2	.86	9	.49	4	.62	7	.62	9	.39	6	.45	9	6.2	2	6.0
	(.10)		(.25)		(.11)		(.14)		(.14)		(.21)		(.22)		(2.0)		
GS	.68	6	.94	7	.49	6	.56	10	.64	5	.40	5	.49	5	6.4	6	6.3
	(.11)		(.17)		(.10)		(.16)		(.11)		(.18)		(.17)		(2.2)		
HPC	.63	7	1.0	2	.45	7	.47	12	.62	6	.35	7	.46	6	7.4	7	6.8
	(.06)		(.00)		(.04)		(.08)		(.04)		(.08)		(.06)		(1.2)		
SPC	.63	8	1.0	2	.45	8	.47	13	.62	7	.35	8	.46	7	7.5	8	7.6
	(.06)		(.00)		(.04)		(.09)		(.04)		(.08)		(.06)		(1.2)		
MMPC	.63	9	1.0	2	.45	9	.47	14	.62	8	.35	9	.46	8	7.5	9	8.5
	(.06)		(.00)		(.04)		(.08)		(.04)		(.08)		(.06)		(1.2)		
Tabu	.61	10	.40	10	.36	10	.69	1	.38	10	.10	10	.10	10	7.9	10	8.9
	(.10)		(.15)		(.14)		(.08)		(.14)		(.21)		(.21)		(1.9)		
H2PC	.57	11	.33	11.5	.30	11	.67	3	.32	11	.00	11	.00	11	8.6	11	10.1
	(.07)		(.11)		(.10)		(.06)		(.10)		(.16)		(.16)		(1.5)		
MMHC	.57	12.5	.33	13.5	.30	12.5	.67	4.5	.32	12.5	.00	12.5	.00	12.5	8.6	12.5	11.6
	(.07)		(.11)		(.10)		(.07)		(.10)		(.16)		(.16)		(1.5)		
RSMAX2	.57	12.5	.33	13.5	.30	12.5	.67	4.5	.32	12.5	.00	12.5	.00	12.5	8.6	12.5	11.6
	(.07)		(.11)		(.10)		(.07)		(.10)		(.16)		(.16)		(1.5)		
HC	.55	14	.33	11.5	.29	14	.65	6	.31	14	-0.02	14	-0.02	14	9.0	14	12.7
	(.08)		(.12)		(.11)		(.07)		(.11)		(.17)		(.17)		(1.6)		

Values in parentheses indicate standard deviations.

F-IAMB: Fast-IAMB; GS: Grow-Shrink; H2PC: Hybrid HPC; HC: Hill-Climbing; HPC: Hybrid Parents and Children; IAMB: Incremental association Markov Blanket; IAMB-F: IAMB with False Discovery Rate Correction; I-IAMB: Interleaved IAMB; MMHC: Max-Min Hill-Climbing; MMPC: Max-Min Parents and Children; PCS: Peter-Clark Stable; RSMAX2: Restricted Maximization; SPC: Semi-Interleaved HITON-PC; Tabu: Tabu search

ACC: Accuracy; SEN: Sensitivity; PRE: Precision; SPE: Specificity; F1: F1 Score; CK: Cohen's Kappa; MCC: Matthews Correlation Coefficient; SHD: Directed Structural Hamming Distance; R: Rank

Table S20. Algorithm performance for DAG_5 with sample size n = 2500.

	ACC	R_{ACC}	SEN	R_{SEN}	PRE	R_{PRE}	SPE	R_{SPE}	F1	R_{F1}	CK	R_{CK}	MCC	R_{MCC}	SHD	R_{SHD}	Overall Rank
PCS	.68	1	.85	8	.49	1	.60	5	.62	1	.38	1	.42	1	6.4	1	2.4
	(.13)		(.16)		(.11)		(.14)		(.13)		(.22)		(.25)		(2.6)		
F-IAMB	.58	4	.92	4	.43	3	.44	11	.58	2	.28	3	.37	2	8.3	4	4.1
	(.14)		(.17)		(.10)		(.21)		(.11)		(.19)		(.18)		(2.7)		
IAMB	.58	4	.92	5.5	.43	4.5	.44	9.5	.58	3.5	.28	4.5	.37	3.5	8.3	4	4.9
	(.14)		(.17)		(.10)		(.21)		(.11)		(.19)		(.18)		(2.7)		
I-IAMB	.58	4	.92	5.5	.43	4.5	.44	9.5	.58	3.5	.28	4.5	.37	3.5	8.3	4	4.9
	(.14)		(.17)		(.10)		(.21)		(.11)		(.19)		(.18)		(2.7)		
IAMB-F	.61	2	.85	9	.43	2	.50	7	.56	8	.28	2	.35	9	7.9	2	5.1
	(.13)		(.22)		(.11)		(.20)		(.13)		(.22)		(.22)		(2.7)		
GS	.58	6	.92	7	.43	6	.44	8	.57	5	.28	6	.36	7	8.3	6	6.4
	(.13)		(.17)		(.10)		(.21)		(.11)		(.19)		(.18)		(2.7)		
SPC	.54	8	1.0	2	.40	7	.34	12	.57	6	.24	7	.37	5	9.2	8	6.9
	(.07)		(.00)		(.04)		(.10)		(.04)		(.08)		(.07)		(1.5)		
MMPC	.54	9	1.0	2	.40	8	.34	13	.57	7	.24	8	.36	6	9.3	9	7.8
	(.07)		(.00)		(.04)		(.10)		(.04)		(.08)		(.07)		(1.4)		
Tabu	.55	7	.36	10	.30	10	.63	1	.34	10	-0.00	10	-0.00	10	9.0	7	8.1
	(.08)		(.12)		(.10)		(.07)		(.10)		(.17)		(.17)		(1.7)		
HPC	.52	13	1.0	2	.39	9	.32	14	.56	9	.22	9	.35	8	9.5	13	9.6
	(.07)		(.00)		(.04)		(.10)		(.04)		(.08)		(.07)		(1.4)		
MMHC	.53	10.5	.32	13.5	.27	11.5	.62	2.5	.30	11.5	-0.05	11.5	-0.05	11.5	9.3	10.5	10.4
	(.06)		(.09)		(.08)		(.06)		(.08)		(.13)		(.13)		(1.2)		
RSMAX2	.53	10.5	.32	13.5	.27	11.5	.62	2.5	.30	11.5	-0.05	11.5	-0.05	11.5	9.3	10.5	10.4
	(.06)		(.09)		(.08)		(.06)		(.08)		(.13)		(.13)		(1.2)		
H2PC	.53	12	.32	11.5	.27	13	.62	4	.29	13	-0.06	13	-0.06	13	9.4	12	11.4
	(.07)		(.09)		(.08)		(.06)		(.08)		(.13)		(.13)		(1.3)		
HC	.52	14	.32	11.5	.26	14	.60	6	.29	14	-0.07	14	-0.07	14	9.7	14	12.7
	(.08)		(.10)		(.08)		(.08)		(.08)		(.15)		(.15)		(1.6)		

Values in parentheses indicate standard deviations.

F-IAMB: Fast-IAMB; GS: Grow-Shrink; H2PC: Hybrid HPC; HC: Hill-Climbing; HPC: Hybrid Parents and Children; IAMB: Incremental association Markov Blanket; IAMB-F: IAMB with False Discovery Rate Correction; I-IAMB: Interleaved IAMB; MMHC: Max-Min Hill-Climbing; MMPC: Max-Min Parents and Children; PCS: Peter-Clark Stable; RSMAX2: Restricted Maximization; SPC: Semi-Interleaved HITON-PC; Tabu: Tabu search

ACC: Accuracy; SEN: Sensitivity; PRE: Precision; SPE: Specificity; F1: F1 Score; CK: Cohen's Kappa; MCC: Matthews Correlation Coefficient; SHD: Directed Structural Hamming Distance; R: Rank

Table S21. Mean changes in MCC, F1, and SHD after hyperparameter tuning across all simulation conditions.

Algorithm	ΔMCC	ΔF1	ΔSHD
HC	.162	.105	-1.061
IAMB-F	.031	.022	-.319
PCS	.029	.021	-.342
I-IAMB	.022	.019	-.446
IAMB	.022	.019	-.446
GS	.022	.019	-.445
F-IAMB	.021	.019	-.442
MMPC	.020	.018	-.462
SPC	.020	.018	-.458
HPC	.018	.016	-.409
Tabu	.015	.012	-.117
MMHC	.008	.003	-.115
RSMAX2	.008	.003	-.115
H2PC	.006	.002	-.091

Values represent mean tuned-minus-default differences across the 20 DAG–sample-size conditions. Positive ΔMCC and ΔF1 and negative ΔSHD indicate improvement.

Table S22. Mean solution time (ms per dataset) of the Bayesian network learning algorithms across sample sizes.

Algorithm	n=200	n=500	n=1000	n=2500	Overall
**SPC**	1.50	1.67	2.01	2.86	2.01
**IAMB**	1.76	1.98	2.44	3.43	2.40
**GS**	1.86	2.11	2.58	3.69	2.56
**MMPC**	1.97	2.21	2.55	3.74	2.62
**I-IAMB**	1.96	2.21	2.68	3.78	2.66
**F-IAMB**	2.38	2.55	2.90	3.76	2.90
**PCS**	2.62	2.66	2.90	3.54	2.93
**HC**	1.15	1.56	2.44	7.60	3.19
**IAMB-F**	3.52	3.90	4.44	5.97	4.46
**RSMAX2**	2.52	3.03	3.89	8.78	4.56
**MMHC**	3.04	3.59	4.49	9.63	5.19
**HPC**	3.88	4.79	5.77	7.66	5.52
**H2PC**	5.06	6.13	7.66	13.90	8.19
**Tabu**	3.17	4.48	7.22	23.49	9.59

Values represent averages across the five DAG structures. For each DAG–sample-size condition, solution time was measured over three runs, and the median of the three elapsed times was used.

Table S23. Cross-software comparison of PC performance between bnlearn and TETRAD.

n	bnlearn MCC	TETRAD MCC	bnlearn F1	TETRAD F1	bnlearn SHD	TETRAD SHD
**200**	0.389	0.378	0.607	0.601	4.669	4.755
**500**	0.474	0.469	0.649	0.646	4.464	4.495
**1000**	0.509	0.497	0.658	0.65	4.527	4.604
**2500**	0.454	0.437	0.609	0.598	5.464	5.538
**Overall**	0.456	0.445	0.631	0.624	4.781	4.848
